# Advances on Hormones and Steroids Determination: A Review of Voltammetric Methods since 2000

**DOI:** 10.3390/membranes12121225

**Published:** 2022-12-02

**Authors:** Joanna Smajdor, Beata Paczosa-Bator, Robert Piech

**Affiliations:** Faculty of Material Sciences and Ceramics, AGH University of Science and Technology, av. Mickiewicza 30, 30-059 Kraków, Poland

**Keywords:** hormones, steroids, electrochemistry, voltammetry

## Abstract

This article presents advances in the electrochemical determination of hormones and steroids since 2000. A wide spectrum of techniques and working electrodes have been involved in the reported measurements in order to obtain the lowest possible limits of detection. The voltammetric and polarographic techniques, due to their sensitivity and easiness, could be used as alternatives to other, more complicated, analytical assays. Still, growing interest in designing a new construction of the working electrodes enables us to prepare new measurement procedures and obtain lower limits of detection. A brief description of the measured compounds has been presented, along with a comparison of the obtained results.

## 1. Introduction 

Steroids are organic compounds, derivatives of the cyclic hydrocarbon sterane, composed of four conjugated homocyclic rings. It is comprised of three cyclohexanes and one cyclopentane ring. Individual rings are marked with consecutive letters of the alphabet, and individual carbon atoms are numbered [1]. Depending on the type of steroid, this skeleton can be extended in various ways with additional carbon atoms, creating various systems, such as the systems of estran, androstane, pregnane, cholate and cholestane. Various functional groups, such as OH, CHO, COOH or CO, can be attached to these systems, changing their biological activity to a large extent. Depending on the function and use, steroids can be divided into five main groups, which include sterols, corticosteroids, anabolic steroids, sex hormones and prohormones. In medical therapy, steroids are used in a wide variety of diseases, including topical conditions, inflammation, autoimmune diseases, allergies or cancer treatment [2,3,4,5]. Steroids, due to their immunosuppressive properties, are also commonly used after organ transplantation to reduce the risk of rejecting an organ [6,7,8]. Apart from its beneficial properties, steroids taken without proper supervision may also cause serious health conditions, such as hypertension, glaucoma, osteoporosis or headaches [9,10,11].

Hormones are organic substances that play a regulatory role in organisms. They are most often produced by the endocrine glands (less often by endocrine tissues) and then transported through the blood to the target cells. Hormone binding by a receptor located on the surface or inside the cell triggers a series of reactions that stimulate or inhibit specific metabolic processes in order to maintain homeostasis of the entire body [12]. There are several different criteria for classifying hormones: due to their place of formation, the chemical structure, mechanism of action, or exerted final effect. Due to the chemical structure, four main groups of hormones can be distinguished, such as peptide and protein hormones, amino acid derivative hormones, steroid hormones and fatty acid analogs [13]. It is known that hormones also affect the functioning of the immune system and even behavior. Therefore, diseases resulting from hormonal disorders have serious consequences on health and can cause multi-organ symptoms. Not only are hormones produced in our body glands, but they are also an important part of the common medicines used in the treatment of hypothyroidism, polycystic ovaries syndrome, breast and prostate cancer, adult acne conditions and during hormone replacement therapy [14,15,16].

By considering the importance of the steroid and hormone families, numerous attempts have been made through the years in order to detect them with high sensitivity using different analytical techniques. Most of the biological samples containing steroids and hormones are analyzed using gas or liquid chromatography coupled with the mass spectrometer [17,18,19,20,21,22]. Determination of the analyte by using these techniques is very accurate; however, the whole analytical procedure is expensive, time-consuming and often requires long and complicated sample pretreatment and specialized skills of the analyst. Considering these disadvantages, electrochemical methods and the development of new electrochemical sensors are gaining much attention lately due to their simplicity, high sensitivity, easiness and low financial outlay. Electroanalysis is one of the analytical methods that help to detect and quantify the analyte in an aqueous solution. Electroanalytical methods, such as voltammetry and polarography, are based on registering the current vs. voltage relation on a voltammogram or polarogram. Voltammetric and polarographic measurements are characterized by potential, which is related to the qualitative properties of the analyte, and the current, which is related to the qualitative amount of the analyte in the measurement cell. The selectivity of the electrochemical methods depends on the accessible potential range for the chosen electrode, the number of compounds in the sample and the half-width of each signal. The choice of optimal techniques depends on several factors, such as the nature of the analyte, the type and material of the electrode and the choice of supporting electrolyte. Specifically, the size and morphology of the working electrode can be very crucial to the analytical response of the system.

This review aims to describe the possibilities of the electrochemical hormones and steroid determination assays that can be successfully applied for their highly sensitive measurements. It is impossible to quote all the papers concerning this topic; thus, only selected manuscripts containing voltammetric, polarographic and amperometric methods are cited. The authors focused on the most recent positions published since 2000.

## 2. Electrochemical Techniques used for Hormones and Steroids Determination

Electrochemistry is a well-established analytical branch and is widely used for the determination of pharmaceutical compounds. The most popular electrochemical techniques used in hormone and steroid analyses are voltammetry and polarography. Notably, voltammetry, which is considered a fast, accurate and high sensitive method, is a willingly chosen technique for the routine quality assurance analysis of electroactive analytes. In voltammetry, a few specific techniques used for qualitative and quantitative measurements can be distinguished, such as cyclic voltammetry (CV), linear sweep voltammetry (LSV), differential pulse voltammetry (DPV) or square-wave voltammetry (SWV). What the above have in common is the basis of signal registration, which is based on the application of a specific potential to the working electrode and registration of the current change in the applied potential range. CV studies are usually used to determine the type of transport of the analyte to the surface of the working electrode, which may be limited by adsorption or diffusion. Additionally, the use of the CV technique allows us to distinguish if the electrode process is reversible or irreversible and thus could be a useful tool for explaining the electrode reaction in detail by giving the possibility to calculate the number of electrons exchanged. CV is very rarely used for the quantitative analysis of compounds because of its lower sensitivity in comparison with other voltammetric techniques. For this type of analysis, the DPV and SWV techniques are widely used. Among the voltammetric techniques, DPV and SWV offer much better sensitivity with a more beneficial signal-to-noise factor, which results in the possibility of performing measurements of electroactive compounds at trace levels. Therefore, these two techniques are used for the studies of active substances present in the pharmaceutical products, such as tablets, creams, ointments, injections, etc., and also often provide opportunities to perform measurements in biological samples, such as urine, blood, serum, etc.

A typical electrochemical sensor used during voltammetric measurements comprises a working electrode, an auxiliary electrode (e.g., platinum rod) and a reference electrode (usually a saturated calomel electrode or Ag/AgCl electrode). The most known working electrodes belong to the family of mercury electrodes, with the dropping mercury electrode (DME) and hanging drop mercury electrode (HMDE) as representatives. Mercury is characterized as the most desired, nearly perfectly polarizable electrode material due to its properties, which include its easy renewal and smooth drop surface, which result in high sensitivity and broad potential windows in the cathodic range. Due to the mentioned properties, the use of mercury in working electrode construction enables the measurement of a wide variety of electroactive compounds. The main disadvantage of the mercury electrodes is the high toxicity of this element, which requires careful work and the utilization of toxic mercury waste after the measurements. Therefore, solid electrodes as an alternative to mercury electrodes are nowadays preferable due to their non-toxic construction. Solid electrodes, made of materials, such as glassy carbon, graphite, noble metals or diamond-related materials, etc., may be characterized by a wide range of working potentials in the anodic region in comparison to the mercury electrodes. The main disadvantage of such electrodes is the necessity for surface preparation before measurement, based on the use of the polishing powder or chemical pretreatment. Solid electrodes also provide a great opportunity to investigate the influence of the surface modifiers’ application on their working surface, which usually results in the improvement of the parameters such as sensitivity and selectivity and allows us to obtain lower detection limits in comparison with the bare electrode. The most popular modifiers include conducting polymers, metallic and non-metallic nanoparticles, carbon nanomaterials, biological compounds or silica-based materials.

A wide variety of working electrodes based on mercury, glassy carbon, noble metals, carbon paste, pyrolytic graphite, molecularly imprinted polymers, carbon nanotubes, nanocomposites, etc., have been developed for the highly sensitive determination of hormones and steroids since 2000. Most of the methods reported so far required immunosensors, which lower the limits of detection in comparison to the classic constructions, which is the impact of the specific bond between the antibody and the analyte, which is strengthened by the catalytic properties of the nanoparticles used in the sensing component of immunosensors.

## 3. Electrochemical Measurements of Hormones and Steroids

### 3.1. Pituitary Gland Hormones

The pituitary gland is a small, endocrine gland placed at the base of the mammal brain. It is responsible for the secretion of hormones that affects bone growth, blood pressure, energy management and functions of the sex organs. The pituitary gland also controls the thyroid gland’s work and metabolism and affects the reproduction process, levels of electrolytes and temperature regulation [23,24].

Human growth hormone (hGH) is a peptide hormone stored and secreted by the somatotropic cells and is based in the anterior pituitary gland. HGH is one of the crucial factors responsible for human development. It also stimulates the production of insulin-like growth factor 1 (IGF-1) and increases the concentration of glucose and free fatty acids. Its recombinant form—somatotropin—is available by prescription and used in the treatment of growth hormone deficiency [25,26,27]. When considering the electrochemical possibilities of hGH determination, there are not many papers with such assays. The lowest obtained detection limit was expressed in the picogram units, which allows for performing analyses on blood or serum samples. The working electrodes used for the high sensitive determination of hGH comprised either specific hGH antibodies or the receptor membrane was constructed via direct electro-polymerization of aniline on the surface of electrodes in the presence of hGH as a template (Table 1). Additionally, it was proven that, based on the voltammetric measurements, it is possible to determine hGH in spiked human serum and plasma and in saliva samples.

Adrenocorticotropic hormone (ACTH) is a peptide hormone of the pituitary gland, classified as a tropic hormone. It stimulates the adrenal cortex to secrete corticosteroids, mineralocorticoids and androgens. Increasing the concentration of ACTH in the blood is one of the body’s first responses to stress. This hormone indirectly influences the body’s protein, carbohydrate and mineral metabolism and also inhibits cell proliferation. Moreover, it has anti-inflammatory and antiallergic properties. Its synthetic form is used to diagnose or exclude primary and secondary adrenal insufficiency, Addison’s disease, and related conditions [28,29]. In the current literature, only two voltammetric assays of ACTH determination are reported. In both cases, the screen-printed carbon electrode was modified with immobilized anti-ACTH antibodies. The lowest limit of detection obtained using such prepared working electrodes was equal to 18 ng/L (Table 1), and the assays were applied for sensitive ACTH determination in human serum samples.

Prolactin (PRL) is a protein hormone best known as a stimulant of breast milk production in mammals. It is secreted in response to physical activity, eating, nursing or mating. Its secretion is also inhibited in some cells in the course of certain cancers and in the endometrium. Excess prolactin (hyperprolactinaemia), often caused by pituitary adenomas, may be responsible for infertility and amenorrhea-galactorrhea syndrome [30,31,32]. For the voltammetric detection of prolactin, mainly the differential pulse technique has been involved for a highly sensitive prolactin determination. Considering the type of sensors being used as working electrodes, the biggest group comprises immunosensors with specific anti-PRL antibodies placed in the receptor layer. The detection limit obtained with this type of sensor is about 4 pg/mL. Apart from the immunosensors, the hanging mercury drop electrode and electrodes decorated with gold nanoparticles, conducting polymers or carbon nanomaterials were used for a PRL determination as well, with good sensitivity and detection limits occurring as low as 38.9 pg/ms (Table 1). Prolactin was determined using voltammetric assays in the samples, such as human urine, saliva, serum and different pharmaceutical formulations.

Thyroid-stimulating hormone (TSH) is a glycoprotein hormone produced by the pituitary gland and comprises alpha and beta subunits. In humans, it causes an increase in the mass of the thyroid gland, an increase in blood flow through this organ and an increase in the production and secretion of thyroid hormones: thyroxine and triiodothyronine. The regulation of thyrotropin secretion is based on the principle of negative feedback with thyroid hormones; secretion is also inhibited by somatostatin and dopamine and stimulated by thyreoliberin and stress or cold [33,34,35]. Only a few voltammetric assays of TSH determination have been reported sincse 2000. Most of them use specific anti-TSH antibodies immobilized on the surface of the working glassy carbon or gold electrode. Such a modification allows for achieving a detection limit as low as 0.005 µIU mL^−1^, and the proposed biosensors were successfully applied for a highly sensitive TSH determination in serum samples, which confirms the usefulness of the developed method (Table 1). TSH was measured electrochemically in human serum samples and in pharmaceutical formulations (tablets).

Follicle-stimulating hormone (FSH) is a glycoprotein hormone comprising 207 amino acids arranged into two subunits. It is secreted by the anterior pituitary gland in both women and men. Acting together with the luteinizing hormone in women, it stimulates the maturation of ovarian follicles and the production of estrogens, and in men, it controls the function of the testicles. In women, FSH stimulates follicular maturation and the secretion of oestrogens from the follicular cells of the ovaries. It also increases the activity of the aromatase enzyme. In men, it causes enlargement of the seminal tubes, stimulates spermatogenesis and increases the production of the androgen-binding protein necessary for the proper functioning of testosterone. During menopause, due to the extinction of the hormonal activity of the gonads, both women and men have elevated levels of FSH in the blood and, and thus, in the urine. Recent research indicates that follicle-stimulating hormone receptors are found in the cells of many types of cancer. This may be important in the diagnosis of neoplasms and allow the creation of drugs targeting cells with FSH receptors [36,37,38,39,40]. Considering the current electrochemical methods of FSH determination, only a few assays have been reported since 2000. Two of them are based on the use of the FSH-specific antibodies entrapped in the modifier layer with reduced graphene oxide, thionine, gold nanoparticles and multi-walled carbon nanotubes. The lowest obtained detection limit obtained with the use of such immunosensors was about 0.05 mIU/mL. In another reported voltammetric assay, the molecularly imprinted polymer, designed in the presence of the FSH particle, was used as a receptor layer along with nickel–cobalt oxide and reduced graphene oxide. The FSH detection limit, in this case, was equal to 0.1 pM (Table 1). The FSH hormone was also measured in samples such as whole human blood and human serum using voltammetric assays. 

In Table 1, the electrochemical methods of hGH, ACTH, prolactin, TSH and FSH determination are compared.

**Table 1:** Electrochemical methods of pituitary gland hormones determination.

Analyte	Technique	Working Electrode	Modifier	Medium	Preconcentration Time, s	Detection Limit	Linear Range	Source
Human Growth Hormone	DPV	SPCE	AuNP/PEDOT/CNT/anti-hGH	0.1 M PBS pH 7.4	n/i	4.4 pg mL^−1^	0.005–1000 ng mL^−1^	[41]
	SWV	GCE	MIP/Fe_3_O_4_	0.2 M PBS pH 6.92	n/i	0.6 × 10^−10^ g cm^−3^	1.0 × 10^−10^–1.0 × 10^−7^ g cm^−3^	[42]
	SWV	SPAuE	TsMBs–mAbhGH–hGH–pAbhGH– anti-IgG–AP	0.1 M Trizma + 1 mM MgCl_2_ buffer pH 9.0	120	0.005 ng mL^−1^	0.01–100 ng mL^−1^	[43]
Adrenocorticotropic hormone (ACTH)	DPV	SPCE	Strept-AP/Biotin-ACTH/anti-ACTH/APBA	n/i	n/i	40 pg L^−1^	5.0 × 10^−3^ –0.1 ng mL^−1^	[44]
	DPV	SPCE	Strept-AP/Biotin-ACTH/anti-ACTH/APBA	n/i	n/i	18 pg L^−1^	0.025–1.0 pg mL^−1^	[45]
Prolactin	DPV	SPCE	Anti-PRL–streptavidin-MBs (magnetic beads)	n/i	n/i	3.7 ng mL^−1^	10–2000 ng mL^−1^	[46]
	DPV	GCE	GPPD-labeled HRP-anti-PRL	ABS pH 5.0	n/i	0.1 ng mL^−1^	0.5–180 ng mL^−1^	[47]
	DPV	CPE	HRP-Ab/PRL/Ab/TGA/nano-Au/CILE	0.1 M PBS pH 7.0	400	12.5 mIU L^−1^	25.0–2000.0 mIU L^−1^	[48]
	DPV	GCE	AP-anti-PRL–PRL-pPPA/MWCNTs	0.1 M PBS pH 7.2	n/i	3 pg mL^−1^	10^−2^–10^4^ ng mL^−1^	[49]
	DPV	GCE	Graphene/AuNPs	0.01 M PBS pH 7.4	n/i	38.9 pg mL^−1^	100 pg mL^−1^–50 ng mL^−1^	[50]
	DPV	GCE	Anti-PRL/pPPA/MWCNTs	PBS pH 7.4	n/i	4 pg mL^−1^	10^−2^–10^4^ ng mL^−1^	[51]
	DPV	CNT/SPCEs	AuNPs/PEDOT	0.1 M PBS pH 7.4	n/i	0.22 pg mL^−1^	0.1–150 ng mL^−1^	[41]
	SWV	HMDE	-	phosphate buffer pH 6.5	90	n/i	0.089–16.36 ng mL^−1^	[52]
	DPV	GCE	Graphene/SWCNT/AuNPs/CT	5.0 mL DEA + 0.75 mg mL^−1^ α-NP	n/i	47 pg mL^−1^	50–3200 pg mL^−1^	[53]
TSH	SWV	GCE	azo compound film	0.1 M phosphate buffer pH 8.0	n/i	0.04 μIU mL^−1^	0.2–20.0 μIU mL^−1^	[54]
	DPV	Au	anti-TSH/AuNP-GO	0.1 M ABS + 6 mM H_2_O_2_ pH 6.0	n/i	0.005 µIU mL^−1^	0.01–20 µIU mL^−1^	[55]
	DPV	Au	GPG-labeled HRP-Ab_2_	0.1 M ABS + 6 mM H_2_O_2_ pH 5.0	n/i	0.005 µIU mL^−1^	0.01–20 µIU mL^−1^	[56]
	DPV	CPE	AuNPs/anti-TSH	0.1 M PBS pH 7.0 + 5.0 mM OAP + 1 mM H_2_O_2_	n/i	0.1 ng mL^−1^	0.2–90.0 ng mL^−1^	[57]
FSH	DPV	SPE	r-GO/thionine Thi/Au NPs/anti- FSH	0.1 M PBS	n/i	1 mIU mL^−1^	1–100 mIU mL^−1^	[58]
	DPV	SPE	r-GO/MWCNTs/ Thi/Au NPs/anti- FSH	0.1 M PBS pH 7.4	1500	0.05 mIU mL^−1^	1–250 mIU mL^−1^	[59]
	LSV	indium tin oxide (ITO)	FSH-MIP/NiCo_2_O_4_/rGO/	0.1 M PBS pH 8.5	n/i	0.1 × 10^−12^ M	0.1 × 10^−12^–1 × 10^−6^ M	[60]

### 3.2. Adrenal Gland Hormones

The adrenal gland is a paired, small endocrine gland located retroperitoneally at the upper pole of the kidney. The adrenal gland comprises cortical and spinal parts, which differ in structure and function. The cortex is the main mass of the gland (80% to 90% of the entire adrenal gland). The cortex produces steroids, which can be divided into three subgroups: mineralocorticosteroids, of which aldosterone has the strongest effect; glucocorticosteroids, the most important of which is cortisol and sex hormones (androgens). The adrenal medulla produces catecholamines. It constantly secretes small amounts of adrenaline into the blood, while all emotional states suddenly release large amounts of it into the blood. Small amounts of norepinephrine are also produced in the adrenal medulla. Hormones secreted by the adrenal cortex maintain the body’s water and mineral balance (aldosterone), help in situations of long-term stress and increase blood glucose levels [12,13,61].

Adrenaline (epinephrine) is an animal hormone and a catecholamine neurotransmitter produced by the endocrine glands of the nerve crest and is secreted at the end of sympathetic nervous system fibers. The term adrenaline is used interchangeably with epinephrine, as both terms refer to exactly the same substance. Adrenaline is also known as the 3xF hormone—the hormone of fear, fight and flight. Adrenaline plays a decisive role in the stress mechanism, the rapid response of the human body and vertebrate animals to a threat, and is manifested by an accelerated heartbeat, increase in blood pressure, bronchial and laryngeal expansion, dilatation of the pupils, etc. In addition, adrenaline regulates the level of glucose in the blood by increasing the breakdown of glycogen into glucose in the liver (glycogenolysis). Adrenaline is also found in plants. Its pharmacological significance is limited due to the low durability of the hormone. Adrenaline is used in cases of cardiac arrest regardless of the mechanism. It has the effect of stimulating the contractility of the heart muscle, improving the conduction of stimuli in the heart, as well as improving the effectiveness of electrical defibrillation. Adrenaline, given in the case of anaphylaxis, quickly relieves the symptoms of an acute allergic reaction. It causes the blood vessels to contract rapidly, which raises blood pressure. The smooth muscles of the bronchi, larynx and throat also relax, which makes breathing easier. Epinephrine also reduces swelling around the mouth and face. Adrenaline is the first-line drug of choice for the treatment of anaphylaxis and the second-line drug for the treatment of cardiogenic shock. It is also used in cases of bronchial asthma attacks and acute allergic reactions when the administration of other drugs does not help and the disease becomes life-threatening. Adrenaline is also used in laryngology and dentistry. It is sometimes used, for example, to reduce bleeding, as it strongly narrows blood vessels [62,63,64,65].

Noradrenaline (norepinephrine) is an organic chemical compound from the group of catecholamines, classified both as a neurotransmitter and hormone, and is secreted in the adrenal medulla and locus coeruleus, usually together with adrenaline. Norepinephrine mobilizes the brain and body to act. Its secretion is lowest during sleep and increases by 180% when awake. It achieves much higher levels in stressful and dangerous situations (fight-or-flight response). In the brain, norepinephrine increases agitation and alertness, supports wakefulness, enhances remembering and recalling and enables concentration, as well as increases anxiety and fear, the excess of which leads to anxiety disorders. In the rest of the body, norepinephrine speeds up the heart rate and increases blood pressure, releases stored glucose, increases blood flow to the skeletal muscles, reduces blood flow to the digestive system, and inhibits bladder emptying and motor activity in the gastrointestinal tract. Norepinephrine, as medicine, is injected in cases of critically low blood pressure [12,13,66,67].

In the current literature, there are numerous reports of adrenaline and noradrenaline determination by using popular voltammetric techniques—cyclic voltammetry, differential pulse voltammetry and square-wave voltammetry. Glassy carbon electrodes, along with the carbon paste electrode, were the most popular working electrodes used in the measurements. Almost in each case, they were modified with different materials in order to achieve higher sensitivity and lower limits of detection. The lowest obtained LOD values were equal to 8.7 × 10^−10^ M and 8.7 × 10^−10^ M for adrenaline and norepinephrine, respectively (Table 2). The voltammetric assays for adrenaline determination were tested on real sample analyses, such as body fluids (serum and urine) and pharmaceutical products in the form of injections.

**Figure 1:**
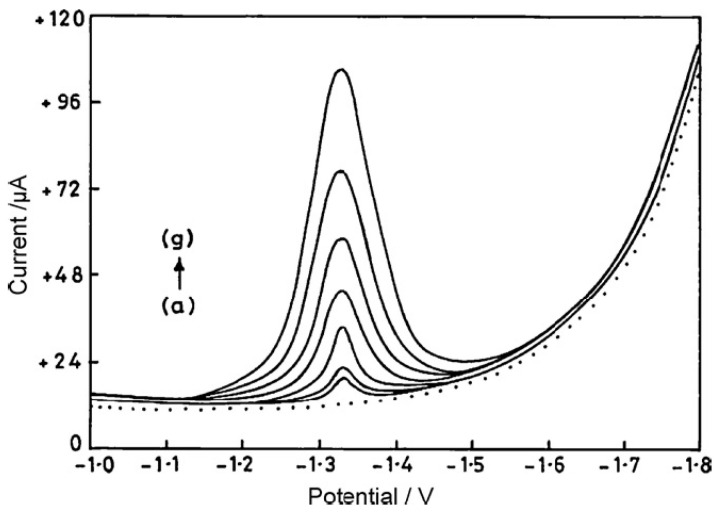
Osteryoung square-wave voltammograms recorded for (a) PBS (background) at the bare EPPGE (· · ·) and (b) increasing concentration of HC at the bare EPPGE electrode (—). Curves were recorded at (a) 100 nM, (b) 250 nM, (c) 500 nM, (d) 750 nM, (e) 1000 nM, (f) 1500 nM and (g) 2000 nM concentration in PBS of pH 7.2. Reprinted from *A comparison of edge- and basal-plane pyrolytic graphite electrodes towards the sensitive determination of hydrocortisone*, Talanta, Vol 83, Rajendra N. Goyal, Sanghamitra Chatterjee, Anoop Raj Singh Rana, Pages 149–155, 2011, with permission from Elsevier [72].

Cortisol (hydrocortisone) is a natural steroid hormone produced by the band layer of the adrenal cortex. It is the main representative of glucocorticosteroids. It has a wide impact on metabolism and is sometimes called the stress hormone, along with adrenaline. It has an anti-inflammatory effect and retains salt in the body. Cortisol increases blood glucose levels, which is indicated in response to stress. Cortisol also releases amino acids from peripheral tissues and inhibits the rate of their absorption by skeletal muscles, accelerates gluconeogenesis, and finally accelerates the breakdown of fatty acids into ketone bodies. Chronic excess of cortisol in the blood leads to the characteristic displacement of adipose tissue deposits (buffalo neck, full moon face, abdominal obesity, lean limbs), thinning of the skin, the formation of characteristic pink stretch marks, acne and insulin resistance, which is a picture of Cushing’s syndrome [68,69,70,71]. The cortisol electrochemical detection assays, since 2000, were mostly performed on solid electrodes, and glassy carbon and gold electrode in particular. In some reported works, immunosensors with anti-cortisol antibodies were used, which allows for obtaining the lowest detection limit, which was equal to 0.64 pM. In such immunosensors, besides the antibody component of the modifier layer, other components, such as conducting polymers or metal nanoparticles, are used to provide the sensor’s stability and even enhance the electrochemical reaction of the analyte. Cortisol could also be measured using a refreshable mercury film electrode with a low detection limit of 4.8 nM (Table 2). Cortisol determination was also performed in the complex matrices, such as human serum and plasma, whole blood and saliva. Additionally, pharmaceutical formulations, such as tablets, creams and ointments, were tested for the possibility of hydrocortisone measurements with success. Samples of the hydrocortisone calibration graphs are presented in Figure 1 [72].

In Table 2, the electrochemical methods of adrenaline, norepinephrine and cortisol determination are compared.

**Table 2:** Electrochemical methods of adrenal gland hormones determination.

Analyte	Technique	Working Electrode	Modifier	Medium	Preconcentration Time, s	Detection Limit, M	Linear Range, M	Source
Adrenaline	SWV	GC	poly(1-methylpyrrole)	0.1 M PBS pH 4.5	0.1	8.7 × 10^−10^	3.0 × 10^−9^–2.0 × 10^−8^	[73]
	DPV	PIGE	PR	0.1 M PBS pH 7.0	n/i	8.0 × 10^−7^	3.0 × 10^−6^–90.0 × 10^−6^	[74]
	SWV	CPE	CuFe_2_O_4_/ILs	0.1 M PBS pH 7.4	n/i	0.07 × 10^−6^	0.1 × 10^−6^–400 × 10^−6^	[75]
	SWV	CPE	PtNP/IL/LAC	0.1 M PBS pH 6.5	n/i	2.9 × 10^−7^	10.0 × 10^−7^–2.1 × 10^−4^	[76]
	DPV	CPE	2,2’-[1,2-Ethanediylbis(nitriloethylidyne)]-bishydroquinone	0.1 M PBS pH 7.0	n/i	2.2 × 10^−7^	7.0 × 10^−7^–1.2 × 10^−3^	[77]
	Amperometry	CPE	GP/mineral oil/polyphenol oxidase	0.1 M acetate buffer pH 4.4	0	1.5 × 10^−5^	5.0 × 10^−5^–3.5 × 10^−4^	[78]
	SWV	CPE	GP–Nujol–peroxidase—CHIT	0.1 M PBS pH 7.0	n/i	4.0 × 10^−7^	2.0 × 10^−6^–1.1 × 10^−4^	[79]
	SWV	CPE	GP–Nujol–BMIPF_6_–peroxidase—CHIT	0.1 M PBS pH 7.0	n/i	2.3 × 10^−7^	9.9 × 10^−7^–1.2 × 10^−4^	[79]
	CV	CPE	GP–binder–poly(isonicotinic acid)	0.1 M PBS pH 5.3	n/i	1.0 × 10^−6^	5.0 × 10^−6^–1.0 × 10^−4^	[80]
	DPV	GC	Poly (eriochrome Black T)	0.05 M PBS pH 3.5	n/i	3.0 × 10^−7^	2.5 × 10^−6^–5.1 × 10^−5^	[81]
	DPV	GC	5,5-ditetradecyl-2-(2-trimethyl-ammonioethyl)-1,3-dioxane bromide	0.1 M PBS pH 6.0	n/i	1.0 × 10^−8^	1.0 × 10^−8^ –1.0 × 10^−4^	[82]
	DPV	Au	Meso-2,3-dimercaptosuccinic	0.1 M PBS pH 7.7	n/i	5.3 × 10^−8^	4.0 × 10^−4^–4.0 × 10^−3^	[83]
	CV	SPE	-	0.1 M H_2_SO_4_ + 0.01 M KCl pH 1	600	1.3 × 10^−7^	2.9 × 10^−7^–1.0 × 10^−4^	[84]
	CV	GC	Caffeic acid	0.1 M PBS pH 7.7	n/i	2.0 × 10^−7^	2.0 × 10^−6^–8.0 × 10^−5^	[85]
	CV	AuE	AuNP/DTT	0.1 M PBS pH 7.0	n/i	6.0× 10^−8^	1.0 × 10^−7^–8.0 × 10^−4^	[86]
	SWV	AuE	Triazole	0.1 M B-R buffer pH 4.4	180	1.0 × 10^−8^	1.0 × 10^−7^–1.0 × 10^−5^	[87]
	DPV	CPE	Laccase-peroxidase	0.1 M PBS pH 6.0	60	2.5 × 10^−8^	6.1 × 10^−6^-1.0 × 10^−4^	[88]
	DPV	GCE	HT/MWCNT	0.1 M PBS pH 7.0	n/i	0.024 × 10^−6^	0.2 × 10^−6^–319.7 × 10^−6^	[89]
	SWV	BDDFE	-	0.5 M HClO_4_	n/i	0.21 × 10^−6^	0.7 × 10^−6^–60 × 10^−6^	[90]
	DPV	CPE	FePC/MWCNT	PBS pH 7.4	n/i	2.1 × 10^−7^	-	[91]
	DPV	CPE	IL/CNT	PBS pH 7.0	n/i	0.09 × 10^−6^	0.3 × 10^−6^–450 × 10^−6^	[92]
	DPV	GCE	SWCNT/CT/IL	PBS pH 7.0	60	0.09 × 10^−6^	1 × 10^−6^–580 × 10^−6^	[93]
	DPV	GCE	PP/MWCNT	PBS pH 6.0	60	0.04 × 10^−6^	0.1×10^−6^–8 × 10^−6^	[94]
	DPV	AuE	PMP	PBS pH 7.2	n/i	0.1 × 10^−6^	-	[95]
	DPV	GCE	Au/PP	0.1 M PBS pH 7.0	240	0.03 × 10^−6^	0.3 × 10^−6^–21 × 10^−6^	[96]
	DPV	GCE	PT	PBS pH 7.4	n/i	0.3 × 10^−6^	2 × 10^−6^–600 × 10^−6^	[97]
	DPV	GCE	PMP	PBS pH 4.0	n/i	0.17 × 10^−6^	0.75 × 10^−6^–200 × 10^−6^	[98]
	DPV	GCE	Pt-AuNP	PBS pH 7.0	n/i	57 × 10^−6^	63–400 × 10^−6^	[99]
	DPV	CPE	acetylene black	0.5 M H_2_SO_4_ + SDS	70	1.0 × 10^−8^	5.0 × 10^−8^–7.0 × 10^−6^	[100]
	DPV	GCE	IMWCNT	0.15 M PBS pH 7.0	n/i	0.96 × 10^−6^	1.3 × 10^−6^–833.3 × 10^−6^	[101]
	DPV	MIP	-	0.1 M K_4_Fe(CN)_6_ + 0.1 M KNO_3_	n/i	2 × 10^−9^	1 × 10^−9^ –100 × 10^−9^	[102]
	DPV	GCE	RuON	0.1 M PBS pH 7.0	n/i	0.45 × 10^−6^	2.0–65.5 × 10^−6^	[103]
	DPV	GCE	pEDOT-CH_2_OH-MIP/MXene/CNHs	0.1 M phosphate buffer 7.4	420	0.3 × 10^−9^	1 × 10^−9^–60 × 10^−6^	[104]
	DPV	CNTPE	poly(phenylmethanoic acid) PPMAM	0.2 M PBS pH 7.0	60	4.5 × 10^−8^	10–110 × 10^−6^	[105]
	DPV	GCE	Pd/BiVO_4_	PBS	n/i	0.154 × 10^−6^	0.9–27.5 × 10^−6^	[106]
	SWV	GCE	phosphorus-doped microporous carbon spheroidal structures (P-MCSs)	0.1 M PB pH 7.0	n/i	2 × 10^−9^	0.01–2 × 10^−6^	[107]
	DPV	CPE	sodium alpha-olefin sulfonate (SAOS)	0.2 M PBS pH 7.4	n/i	0.14 × 10^−6^	10–70 × 10^−6^	[108]
	DPV	CPE	triiodide ions immobilized in an anion-exchange resin	PBS pH 6.0	n/i	3.9 × 10^−6^	2.0 × 10^−5^–3.1 × 10^−4^	[109]
	DPV	Au	ME/Au SAMs	0.04 M B-R buffer pH 4.0	250	3.3 × 10^−8^	1.0 × 10^−7^–1.0 × 10^−4^	[110]
Norepinephrine	SWV	GCE	TAPP	PBS pH 7.4	n/i	-	1.0 × 10^−6^–5.0 × 10^−5^	[111]
	SWV	Pd	graphene	PBS pH 7.2	n/i	67.4 × 10^−9^	0.0005–0.5 × 10^−3^	[112]
	CV	GCE	graphene	PBS pH 7.0	120	400 × 10^−9^	6.00 × 10^−7^–1.20 × 10^−4^	[113]
	CV	GCE	graphene	PBS pH 4.0	n/i	100 × 10^−9^	0.6–1000 × 10^−6^	[114]
	DPV	GCE	PEDOPA-NFs	0.1 M PBS pH 7.4	n/i	50 × 10^−9^	0.3–10 × 10^−6^	[115]
	DPV	GCE	Polyisonicotinic acid	PBS pH 5.6	n/i	6.0 × 10^−9^	4.0 × 10^−7^–2.0 × 10^−4^	[116]
	CV	GCE	SWCNT	B–R buffer pH 5.72	n/i	6000 × 10^−9^	1.0 × 10^−5^–1.1 × 10^−3^	[117]
	DPV	GCE	C-Ni	PBS pH 7.0	120	60 × 10^−9^	2.0 × 10^−7^–8.0 × 10^−5^	[118]
	CV	Graphite	β-CD/CNT	0.1 M PBS pH 6.0	n/i	5.0 × 10^−7^	1.0 × 10^−6^–3.0 × 10^−4^	[119]
	CV	Au	TLA	PBS pH 5.91	2	2.0 × 10^−6^	4.0 × 10^−5^–2.0 × 10^−3^	[120]
	SWV	EPPGE	MWNT	PBS pH 7.2	n/i	0.90 × 10^−10^	0.5–100 × 10^−9^	[121]
	DPV	CPE	ZrO_2_NPs	0.1 M PBS pH 7.0	n/i	8.9 × 10^−8^	1.0 × 10^−7^–2.0 × 10^−3^	[122]
	CV	GCE	Calix[4]arene crown-4	0.1 M PB pH 6.0	n/i	2.8 × 10^−7^	5.5 × 10^−7^–2.3 × 10^−4^	[123]
	CV	Au	C_60_-[dimethyl-(β-cyclode×trin)]_2_/Nafion	0.1 M PB pH 6.0	n/i	8.0 × 10^−6^	5.0 × 10^−5^–5.8 × 10^−4^	[124]
	SWV	GCE	Poly2,4,6-trimethylpyridine	PBS pH 7.4	n/i	8.0 × 10^−6^	5.0 × 10^−3^–1.0 × 10^−1^	[125]
	CV	GCE	Poly(cresol red)	0.1 M PBS pH 3.0	n/i	2.0 × 10^−7^	3.0 × 10^−6^–3.0 × 10^−5^	[126]
	CV	GCE	Nickel(II) complex	0.1 M PBS pH 7.4	n/i	7.7 × 10^−9^	1.0 × 10^−7^–1.0 × 10^−5^	[127]
	DPV	GCE	Au NPs	PBS pH 7.0	n/i	2.0 × 10^−8^	5.0 × 10^−7^–8.0 × 10^−5^	[128]
	DPV	TiO_2_ NPs—CPE	2,2’-[1,2-butanediylbis(nitriloethylidyne)]-bishydroquinone	pH 8.0	n/i	5.0 × 10^−7^	4.0 × 10^−6^–1.1 × 10^−3^	[129]
	CPV	SPE	PAA-MWCNTs	0.1 M PBS pH 7.5	1500	1.3 × 10^−7^	0–1.0 × 10^−5^	[130]
	SWV	ITO	AuNPs	PBS pH 7.2	n/i	87 × 10^−9^	100 × 10^−9^–25 × 10^−6^	[131]
	DPV	CPE	MCM-41	PBS pH 7.0	n/i	4.0 × 10^−8^	7.0 × 10^−8^–2.0 × 10^−3^	[132]
	DPV	CPE	Molybdenum (VI) complex	PBS pH 7.0	n/i	4.3 × 10^−8^	8.0 × 10^−8^–7.0 × 10^−4^	[133]
	DPV	GCE	Hematoxylin	0.15 M PBS pH 7.0	n/i	1.4 × 10^−7^	5.0 × 10^−7^–2.7 × 10^−4^	[134]
	DPV	CPE	3,4-dihydroxybenzaldehyde-2,4-dinitrophenylhydrazone	0.1 M PBS pH 7.0	n/i	7.7 × 10^−8^	1.0 × 10^−7^–8.0 × 10^−4^	[135]
	DPV	CPE	Ferrocenemonocarboxylic acid (FMC)	0.1 M phosphate buffer pH 7.0	n/i	1.6 × 10^−7^	5.2 × 10^−7^–5.3 × 10^−4^	[136]
	DPV	CPE	Chloranile	0.1 M PBS pH 7.0	n/i	1.12 × 10^−8^	3.0 × 10^−8^ –5.0 × 10^−4^	[137]
	DPV	GCE	Polycalconcarboxylic acid	PBS pH 6.0	n/i	0.1 × 10^−6^	0.63–62.5 × 10^−6^	[138]
	DPV	GCE	Au-NPs/poly(2-amino-2-hydroxymethyl-propane-1,3-diol)	PBS pH 3.0	n/i	0.07 × 10^−6^	1.3–230.1 × 10^−6^	[139]
	CV	GCE	MWCNT/FCo_98_ (cobalt ferrite nanoparticles)	0.1 M PBS pH 7.0	n/i	0.76 × 10^−6^	0.16–1.91 × 10^−3^	[140]
	CV	CPE	poly (rhodamine B)	0.2 M PBS pH 7.4	n/i	1.8 × 10^−6^	20–90 × 10^−6^	[141]
	SWV	GCE	GQDs/AuNPs (quantum dots)	PBS pH 7.0	30	0.15 × 10^−6^	0.5–7.5 × 10^−6^	[142]
	DPV	Au	Cys/CDs/Tyr	0.1 M PBS pH 7.0	n/i	196 × 10^−9^	1–200 × 10^−6^	[143]
	SWV	GCE	P(L-Arg)/ERGO	PBS pH 7.0	n/i	4.22 × 10^−8^	2 × 10^−5^–8 × 10^−7^	[144]
	DPV	CPE	GQDs/IL	0.1 M PBS pH 7.0	n/i	0.06 × 10^−6^	0.2–400 × 10^−6^	[145]
	SWV	PGE	-	0.1 M PBS pH 7.4	n/i	9.92 × 10^7^	2.5 × 10^−4^–2.5 × 10^−6^	[146]
	CV	CPE	MnCr_2_O_4_	0.2 M PBS pH 7.4	n/i	0.034 × 10^−6^	0.3 × 10^−6^–4.5 × 10^−6^	[147]
	DPV	CPE	MWCNTs/CILE	0.1 M PBS pH 7.0	n/i	0.09 × 10^−6^	0.3–450 × 10^−6^	[148]
	SWV	CPE	5-mino-3′,4′-dimethyl-biphenyl-2-ol	0.1 M PBS pH 7.0	n/i	5.9 × 10^−7^	1.2 × 10^−6^–9.0 × 10^−4^	[149]
	DPV	CPE	poly(glutamic acid)	0.2 M PBS pH 7.4	n/i	0.43 × 10^−6^	n/i	[150]
	DPV	CPE	CNT + ferrocene (FC)	0.1 M PBS pH 7.0	n/i	0.21 × 10^−6^	0.47–500.0 × 10^−6^	[151]
	CV	CPE	Tx-100	0.1 M PBS pH 7.0	n/i	5.0 × 10^−6^	0.5 × 10^−4^–2.0 × 10^−4^	[152]
	SWV	CPE	MWNTs/MBIDZCl	0.1 M PBS pH 7.0	n/i	0.08 × 10^−6^	0.2–500 × 10^−6^	[153]
Cortisol (hydrocortisone)	DPV	Hg(Ag)FE	-	Acetate buffer pH 4.2	30	4.8 × 10^−9^	0.02 × 10^−6^–1.2 × 10^−6^	[154]
	SWV	EPPG	-	PBS pH 7.2	-	88 × 10^−9^	100–2000 × 10^−9^	[72]
	SWV	SPE	mAbC	PBS pH 7.4	-	1.7 × 10^−9^	1.7 × 10^−9^–1.2 × 10^−7^	[155]
	CV	Au	BSA/C-Mab/PPAuNP	PBS pH 7.0	-	1 × 10^−12^	1 × 10^−12^–100 × 10^−9^	[156]
	CV	Au	EA/anti-AbC/DTSP	0.05 M PBS pH 7.4	n/i	2.8 × 10^−11^	10 × 10^−12^–100 × 10^−9^	[157]
	DPV	ITo	AbC/NiO	PBS pH 7.0	n/i	0.89 × 10^−12^	2.8 × 10^−12^–27.5 × 10^−3^	[158]
	amperometry	Au	AbC	0.01 M PBS pH 7.5 + glucose	n/i	2.8 × 10^−9^	3.4 × 10^−9^–5.5 × 10^−7^	[159]
	amperometry	SPE	RGO/ AbC	PBS pH 7.4	n/i	2.8 × 10^−10^	2.8 × 10^−10^–5.5 × 10^−7^	[160]
	CV	Au	ZnO/AbC	0.01 M PBS pH 7.4	n/i	1 × 10^−12^	1 × 10^−12^–100 × 10^−9^	[161]
	DPV	GCE	HRP-Strept-Biotin-AbC/AuNPs/MrGO/Nafion	0.1 M pH 7.0 PBS + 2 mM o-PD + 4 mM H_2_O_2_	n/i	1.4 × 10^−10^	2.8 × 10^−10^–2.8 × 10^−6^	[162]
	DPV	CPE	β-cyclodextrin	0.04 M B-R pH 3.0	150	3.7 × 10^−7^	4.2 × 10^−7^–2.5 × 10^−5^	[163]
	DPV	SPE	AbC/ protein A-magnetic beads	0.1 M Trizma + 1 mM MgCl_2_ pH 9.0	n/i	9.7 × 10^−12^	1.4 × 10^−11^–4.1 × 10^−7^	[164]
	SWV	Au	AuNPs/AbC	0.02 mM PBS pH 7.4	n/i	4.4 × 10^−11^	0.14 × 10^−9^–7 × 10^−9^	[165]
	CV	IDE/Cr/Si	AbC /DTSP-SAM	PBS pH 7.4	n/i	2.8 × 10^−11^	2.8 × 10^−11^–2.8 × 10^−8^	[166]
	CV	SPE	MIP-PPy	0.01 M PBS pH 7.4	n/i	1 × 10^−12^	1 × 10^−12^–10 × 10^−6^	[167]
	SWV	CPE	AuNPs/MWCNT/IL	0.1 M Trizma buffer pH 9.0	n/i	2.5 × 10^−7^	2.8 × 10^−7^–3.3 × 10^−4^	[53]
	DPV	GCE	CoO NPs/Naf	0.1 M NaOH pH 12.0	n/i	0.49 × 10^−9^	0.001–9.0 × 10^−6^	[168]
	DPV	SPCE	cortisol-AP/anti-cortisol/APBA	n/i	n/i	1.0 × 10^−10^	2.8 × 10^−10^–1.4 × 10^−6^	[44]
	DPV	GCE	PDA-ERGO polydopamine/rGO	0.1 M acetate buffer pH 5.2	n/i	0.006 × 10^−9^	0.001–50 × 10^−6^	[169]
	CV	SPCE	MIP	K_3_[Fe(CN)_6_]/K_4_[Fe(CN)_6_] + 0.1 M KCl	n/i	1.2 × 10^−9^	1.3–20 × 10^−9^	[170]
	DPV	Nano-porous GCE	-	0.1 M PBS pH 2	n/i	30 × 10^−9^	0.1–42 × 10^−6^	[171]
	CV	Au	BSA/Anti-Cab/Ag@AgO–PANI	PBS pH 7.0 + 0.9% NaCl	n/i	0.64 × 10^−12^	1 × 10^−12^–1 × 10^−6^	[172]
	CV	Pt	Anti-C_ab_	PBS	n/i	2.8 × 10^−9^	2.8 × 10^−9^–2.8 × 10^−8^	[173]
	CV	Si	rGO/Au IDA	0.05 M bicarbonate–carbonate buffer pH 9.6	n/i	2.8 × 10^−9^	0–500 × 10^−6^	[174]

### 3.3. Pancreatic Hormones 

The pancreas is a glandular organ of vertebrates, while the islet pancreas also occurs in other chordates. It takes various forms in different taxa. It can be a compact organ, or it can be scattered among other tissues. It arises from three buds in the intestinal epithelium, one of which disappears in mammals. It consists of two types of tissue: follicular and insular. Pancreatic follicles are exocrine glands that produce many digestive enzymes and break down different types of food. They combine with the secretions of the walls of the exit ducts leading into the intestine to form pancreatic juice. Pancreatic islets diffuse or form a separate organ. There are five types of cells. They function as endocrine glands, secreting, e.g., hormones, such as insulin, somatostatin, and also glucagon in gnathostomatas [175,176,177,178].

Insulin is an anabolic peptide hormone with a systemic effect that plays an essential role in the metabolism of carbohydrates, as well as proteins and fats, secreted by the endocrine part of the pancreas, more specifically by the beta cells of Langerhans islands. The primary task of insulin is to lower blood glucose levels. This happens in four different ways: by increasing glucose transport to insulin-responsive tissues, increasing the use and storage of glucose by tissues, increasing the use of amino acids and increasing fat synthesis. A synthetic form of insulin is commonly used in the treatment of type 1 diabetes, where the pancreatic beta islet cells stop producing this hormone, which in turn causes chronic hyperglycemia [179,180,181,182]. When considering the still-growing amount of diabetes causes and the importance of the accurate systems of direct insulin measurements in the human body, new electrochemical sensors for insulin determination are still demanded. Since 2000, numerous works of voltammetric and amperometric insulin determination methods have been reported. Most of them used glassy carbon electrodes as working electrodes, modified by different types of nanomaterials, such as carbon nanotubes, nickel nanoparticles or reduced graphene oxide. Among the various materials, transition metal oxides and hydroxides have attracted great interest in electrochemical studies because of their excellent electrocatalytic activity toward different compounds. The use of such modifiers allows for obtaining picomolar insulin limits of detection (Table 3). The great interest in insulin determination, therefore, results in the preparation of voltammetric assays for its sensitive measurement in human serum and plasma samples and in injections commonly used by people with diabetes. 

In Table 3, the electrochemical methods of insulin determination are compared.

**Table 3:** Electrochemical methods of pancreatic hormones determination.

Analyte	Technique	Working Electrode	Modifier	Medium	Detection Limit	Linear Range	Source
Insulin	amperometry	CNT-NiCoO_2_ /Nafion	-	0.1 M PBS pH 7.5	0.22 µg mL^−1^	0.1–31.5 µg mL^−1^	[183]
	FIA	CCE	CHN	0.3 M PBS pH 10	0.11 × 10^−9^ M	0.5–15 × 10^−9^ M	[184]
	Amperometry	GCE	IrOx	0.1 mM Na_3_IrCl_6_ + 0.2 M HCl pH 7.4	20 × 10^−9^ M	50–500 × 10^−9^ M	[185]
	Amperometry	CCE	[Ru(bpy) (tpy)CI]PF_6_	0.1 M PBS pH 7.0	0.4 × 10^−9^ M	0.5–850 × 10^−9^ M	[186]
	FIA	CPE	RuOx	0.1 M NaCl + 0.05 M phosphate buffer pH 7.4	50 × 10^−9^ M	100–1000 × 10^−9^ M	[187]
	FIA	GCE	RuOx-CNT	0.05 M PBS pH 7.4	1 × 10^−9^ M	10–80 × 10^−9^ M	[188]
	Amperometry	SPE	MWCNT/NiONPs	0.1 M NaOH pH 13	6.1 × 10^−9^ M	20–260 × 10^−9^ M	[189]
	FIA	GCE	SiC	PBS pH 7.4	0.0033 × 10^−9^ M	0.1–0.6 × 10^−9^ M	[190]
	Amperometry	CPE	Si	PBS pH 2.0	36 × 10^−12^ M	90–1400 × 10^−12^ M	[191]
	FIA	C	RuRDMs	0.2 M PBS pH 7.0	2 × 10^−9^ M	6–400 × 10^−9^ M	[192]
	Amperometry	GCE	CT/CNT	PBS pH 7.4	30 × 10^−9^ M	100–3000 × 10^−9^ M	[193]
	CV	ITO	NiNPs	0.1 M NaOH	10 × 10^−9^ M	1 × 10^−9^–125 × 10^−9^ M	[194]
	CV	CFME	NiNPs/CNTs	0.1 M NaOH	270 × 10^−9^ M	2–20 × 10^−6^ M	[195]
	DPV	GCE	SiO_2_ NPs/Nafion	0.1 M PBS pH 7.35	3.1 × 10^−9^ M	10–50 × 10^−9^ M	[196]
	FIA	GC	CNT	0.05 M PBS pH 7.4	14 × 10^−9^ M	100–1000 × 10^−9^ M	[197]
	FIA	CCE	NiNPs	0.1 M PBS pH 13	2.6 × 10^−12^ M	15–100 × 10^−12^ M	[198]
	CV	GC	rGO	0.1 M PBS pH 7.4	350 × 10^−12^ M	4–640 × 10^−9^ M	[199]
	amperometry	GC	NiO/guanine	PBS pH 7.4	22 × 10^−12^ M	100 × 10^−12^ M–4 × 10^−6^ M	[200]
	amperometry	GCE	Ni(OH)_2_NPs/Nafion-MWCNT	0.1 M NaOH	85 × 10^−9^ M	0–40 × 10^−6^ M	[201]
	SWV	PGE	NiNPs/MB	B-R buffer pH 7.0	33.17 × 10^−9^ M	25–450 × 10^−9^ M	[202]
	CV	TFT microelectrodes	MWCNT	0.05 M PBS pH 7.4	250 × 10^−9^ M	250 × 10^−9^–1.6 × 10^−6^ M	[203]
	FIA	GCE	CoOx	PBS pH 9.0	25 × 10^−12^ M	100 × 10^−12^ M–15 × 10^−9^ M	[204]

### 3.4. Pineal Gland Hormones

The pineal gland is one of the endocrine glands, which lies between the upper mounds of the lamina. The pineal gland cells—pinealocytes—produce the so-called sleep hormone – melatonin, a derivative of tryptophan [205,206,207]. Melatonin and its derivative metabolites are secreted into the cerebrospinal fluid and into the blood. Its secretion is closely related to light stimulation; its presence inhibits the production of this hormone. In mammals, it also has an inhibitory effect on the secretion of gonadotropic hormones, preventing premature sexual maturation. The secretory activity of the pineal gland follows the daily rhythm of changes in lighting and probably affects the rhythmicity of various physiological functions. In mammals, the secretion of the pineal gland is controlled by impulses sent by the eye’s retina. Disturbances in the work of this gland cause disturbance of the circadian rhythm and, in the long term, disturbances in the development of gonads. Melatonin is sold in the form of over-the-counter tablets as a drug to help people fall asleep, who have disturbed circadian rhythms, or for blind patients and those with sleep disorders related to changing time zones (sudden change in time zone syndrome) [208,209,210,211,212].

**Figure 2:**
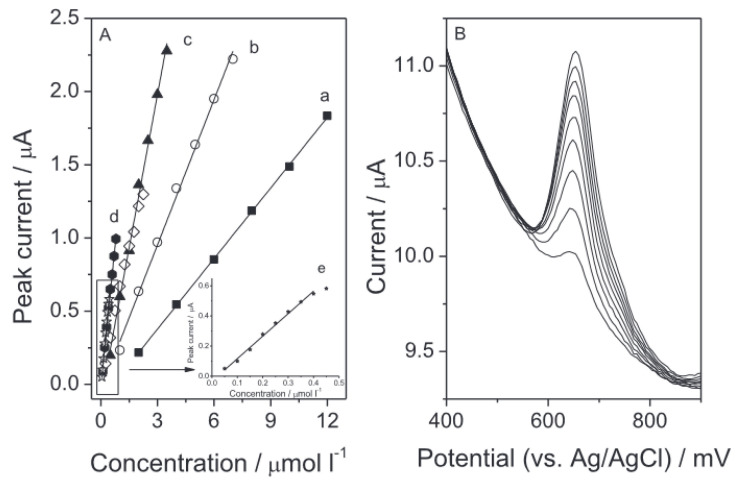
Melatonin calibration graphs (**A**) with corresponding voltammograms (**B**) measured on a glassy carbon electrode modified with carbon black [213]. The following curves in picture A stand for preconcentration times: a: 0 s, b: 3 s, c: 6 s, d: 10 s and e: 20 s, measurements carried out in the 0.1 mol L^−1^ phosphate buffer pH 6.2. Reprinted from *Carbon black as a glassy carbon electrode modifier for high sensitive melatonin determination*, Journal of Electroanalytical Chemistry, Vol 799, Joanna Smajdor, Robert Piech, Magdalena Pięk, Beata Paczosa-Bator, Pages 278–284, 2017, with permission from Elsevier.

A large variety of sensors based on polymers, nanoparticles, carbon-based materials, hybrid arrangements and biomolecules were used for the highly sensitive determination of melatonin. According to the literature, melatonin determination on an unmodified solid electrode has been performed on a boron-doped diamond electrode, carbon paste electrode and glassy carbon electrode thus far. In order to obtain a high sensitivity of the performed measurements, different types of electrode modifications have been developed since then. The most popular include multi-walled carbon nanotubes, palladium nanoparticles, nanorods of ZnO_2_, graphene or carbon black. The lowest detection limit obtained for melatonin was measured using the alternating current voltammetry with a carbon paste electrode as a working electrode, and it was equal to 9 × 10^−11^ M (Table 4). Samples of the melatonin calibration graphs are presented in Figure 2 [213]. Melatonin measurements were performed using voltammetric techniques in the samples, such as human serum and plasma, urine, and in pharmaceutical formulations in the form of tablets. 

In Table 4, the electrochemical methods of melatonin determination are compared.

**Table 4:** Electrochemical methods of pineal gland hormones determination.

Analyte	Technique	Working Electrode	Modifier	Medium	Preconcentration Time, s	Detection Limit, M	Linear Range, M	Source
Melatonin	DPV	GCE	CB/DMF	0.1 M phosphate buffer pH 6.2	45	1.9 × 10^−8^	0.05 × 10^−6^–12 × 10^−6^	[213]
	CV	BDD	-	1 M KCl	n/i	1.03 × 10^−5^	3.4 × 10^−4^–6.8 × 10^−4^	[214]
	SWV	GCE	AHNSA/PdNPs/ErGO	1 M phosphate buffer pH 7.2	n/i	0.09 × 10^−6^	5 × 10^−6^–100 × 10^−6^	[215]
	SWV	BDD	-	0.1 M B-R buffer pH 3.0	60	1.1 × 10^−7^	0.5 × 10^−6^–4.0 × 10^−6^	[216]
	DPV	GCE	-	0.5 M H_2_SO_4_ + 20% methanol pH 2.15	n/i	5.86 × 10^−6^	20 × 10^−6^–80 × 10^−6^	[217]
	amperometry	GCE	MnHCF/PEDOT	0.1 M KCl buffer pH 1.5	3	100 × 10^−6^	100–4600 × 10^−6^	[218]
	DPV	GCE	RGO/RuO_2_	PBS pH 7.0	n/i	0.18 × 10^−6^	2–20 × 10^−6^	[219]
	LSV	GCE	-	0.05 M phosphate buffer pH 3.0	n/i	1.1 × 10^−5^	2.7 × 10^−5^–2.2 × 10^−4^	[220]
	DPV	GCE	3DG/AuNPs	0.04 M PBS pH 7.4	n/i	0.0082 × 10^−6^	0.05-50 × 10^−6^	[221]
	amperometry	CPE	-	0.1 M HClO_4_	0	8 × 10^−9^	10^−8^–10^−5^	[222]
	DPV	CILE	MWCNTs–CoHNPs	0.01 M PBS pH 7.5	90	0.004 × 10^−6^	0.01–50 × 10^−6^	[223]
	SWV	GPT/WPE	-	0.1 M phosphate buffer pH 7.4	n/i	32.5 × 10^−9^	0.8–100 × 10^−6^	[224]
	SWV	SPE	-	0.1 M phosphate buffer pH 5.0	n/i	25.8 × 10^−9^	0.25–75 × 10^−6^	[225]
	LSV	GCE	MWNTs-DHP	0.01 M PBS pH 7.5	180	0.02 × 10^−6^	0.08–10 × 10^−6^	[226]
	OSWSV	AGCE	-	0.04 M B-R buffer pH 6.7	120	0.05 × 10^−6^	0.8–10 × 10^−6^	[227]
	SWV	CPE	Graphene/Fe_2_O_3_	B–R buffer pH 5.0	n/i	8.4 × 10^−9^	0.02–5.8 × 10^−6^	[228]
	ACV	CPE	-	0.1 M HClO_4_	600	9.0 × 10^−11^	1.0 × 10^−10^–1.0 × 10^−9^	[229]
	SWV	HMDE	-	acetate buffer pH 5.0	30	3.1 × 10^−10^	1 × 10^−9^–1 × 10^−7^	[230]
	SWV	GCE	GR/AHNSA/MM	PBS pH 7.2	240	60 × 10^−10^	0.05–100 × 10^−6^	[231]
	SWV	Gr-AV	-	0.5 M McIlvaine buffer solution pH 7.0	n/i	0.49 × 10^−6^	10–100 × 10^−6^	[232]
	DPV	CPE	PdNP@Al_2_O_3_	0.1 M PB pH 7.0	n/i	21.6 × 10^−9^	6 × 10^−9^–1.4 × 10^−3^	[233]
	DPV	GCE	-	B–R buffer pH 4.3	n/i	1.48 × 10^−6^	10–500 × 10^−6^	[234]
	SWV	Au	acetylene black NPs-chitosan (AB-C)	0.1 M PBS pH 7.0	0	1.9 × 10^−6^	2 × 10^−5^–4.5 × 10^−4^	[235]
	DPV	SPE	MWCNTs	50 mM phosphate buffer pH 7.6	n/i	1.1 × 10^−6^	0.005–3 × 10^−3^	[236]
	CV	SPE	graphene	0.1 M PBS pH 7.0	n/i	0.87 × 10^−6^	1–300 × 10^−6^	[237]

### 3.5. Ovarian Hormones

The ovarium is a gonad found in the females of most animals (except for sponges). Usually, a paired organ is found in females, which is the developmental equivalent of the testicles. The ovaries lie inside the peritoneal cavity at the side walls of the pelvis on the posterior surface of the broad ligaments of the uterus, to which they are attached by the short mesentery. The ovaries serve a dual purpose: the production of eggs and the secretion of female sex hormones (estrogens, progesterone, relaxin and androgens). From the moment a woman reaches sexual maturity until the end of her reproductive function (menopause), the so-called Graaf’s follicle contains an egg. A follicle that is ripe to rupture is 1–1.5 cm in diameter, and the egg cell is about 0.2 mm. Follicle maturation occurs under the influence of the follicle-stimulating hormone, and under the influence of the luteinizing hormone, the amount of fluid in the follicle increases, and finally, it ruptures. When the follicle ruptures, the egg enters the fallopian tube, and the rest of the follicle produces a red body—and a corpus luteum from it—which releases the progesterone necessary to implant a fertilized egg into the uterine mucosa [238,239,240].

Estrogens are a group of sex hormones that include the three main forms of estrogen naturally occurring in women: estrone (E1), estradiol (E2) and estriol (E3), as well as the estrogen produced only during pregnancy, estetrol (E4). Estrogens are called female hormones, and they play the most important role in the female body, but they are also essential for men, where a deficiency in the testes can cause infertility. Estrogens are steroidal hormones; they differ in the number and arrangement of the hydroxyl groups. They affect many features and functions of the body, especially the female body. They are mainly responsible for the development of second or third-order sexual characteristics of a woman’s body, regulation of the menstrual cycle, lipid and calcium metabolism or increasing blood clotting. During the menstrual cycle, the estradiol levels fluctuate, which produces specific physiological effects that prepare the uterus to receive and facilitate a fertilized egg, mainly by causing endometrial growth [241,242,243,244].

**Figure 3:**
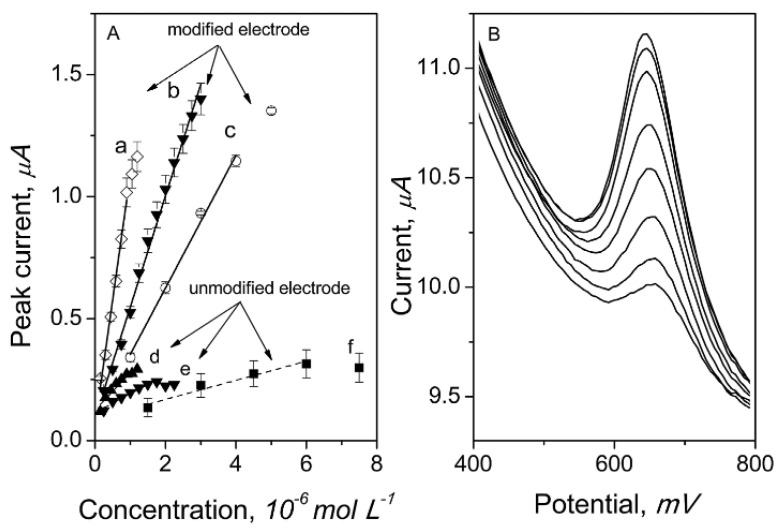
Estradiol calibration graphs (**A**) with corresponding voltammograms for the preconcentration time of 60 s (**B**) measured on a glassy carbon electrode modified with carbon black [245]. The following curves in picture A stand for preconcentration times: a: 60 s, b: 20 s and c: 5 s, measurements carried out in the 0.1 mol L^−1^ phosphate buffer pH 6.2. Reprinted from *Glassy carbon electrode modified with carbon black for sensitive estradiol determination by means of voltammetry and flow injection analysis with amperometric detection*, Analytical Biochemistry, Vol 544, Joanna Smajdor, Robert Piech, Martyna Ławrywianiec, Beata Paczosa-Bator, Pages 7–12, 2018, with permission from Elsevier.

In the current literature, the topic of estrogen-compound determination is still gaining popularity due to being considered as a group of one of the biggest environmental pollutants, which has the ability to interfere with the endocrine system. In particular, the electrochemical methods have received extensive attention from researchers because of its sensitivity and short time of analysis. Square wave voltammetry and differential pulse voltammetry are especially useful for the detection of small amounts of estrogen. The lowest detection limits for E1, E2 and E3 were equal to 0.23 pM, 0.54 pM and 0.5 nM, respectively (Table 5). In order to check the utility of the proposed methods for routine quality control analyses, the measurements were performed in samples, such as water (tap water, wastewater and surface water) human urine, blood, serum, plasma, animal tissues and milk. Samples of the estradiol calibration graphs are presented in Figure 3 [245].

Progesterone (lutein) is a female sex hormone with a steroid structure, produced mainly by the luteal cells in the luteal phase. It is one of the most important hormones secreted by the ovaries. This hormone enables the embryo to implant in the uterine mucosa and maintains the pregnancy. If pregnancy does not occur, progesterone secretion is reduced, and corpus luteum luteolysis occurs. The rapid reduction in blood progesterone levels results in a controlled shedding of the lining of the womb (menstruation). After fertilization, progesterone is initially secreted by the corpus luteum, and in the 14–18 weeks of pregnancy (in humans), it is also produced by the placenta. In the female body, progesterone works through the appropriate receptors located, among others, in the uterus, mammary glands, CNS (central nervous system) and pituitary gland. Progesterone acts synergistically with estrogens on the mammary gland, stimulating the growth of the glandular cells and ductal epithelium and participating in the expression of the receptors necessary for lactation. Other metabolic effects of progesterone include an increasing body temperature, stimulating breathing, lowering the concentration of amino acids in the blood serum, normalization of blood glucose levels, and antiandrogenic activity consisting in the activity’s inhibition of 5-alpha reductase, which transforms testosterone into dihydrotestosterone. The fall in progesterone levels after childbirth causes mood swings, known as postnatal depression [246,247,248,249]. Many electrochemical techniques of progesterone determination are based on the modification of solid electrodes with a layer comprising progesterone-specific antibodies or aptamers. The use of such immunosensors allows for obtaining a progesterone limit of detection as low as 1.86 pM. Additionally, modifiers, such as metal oxides, multi-walled carbon nanotubes, conducting polymers or other carbon nanomaterials, are commonly used in progesterone determination assays. The limits of detection, obtained in the literature since 2000, allow for the measurement of progesterone in samples, such as human serum, plasma, milk, and in pharmaceutical formulations in the form of injections.

In Table 5, the electrochemical methods of estradiol, estriol, estrone and progesterone determination are compared.

**Table 5:** Electrochemical methods of ovarian hormone determination.

Analyte	Technique	Working Electrode	Modifier	Medium	Preconcentration Time, s	Detection Limit	Linear Range	Source
Estradiol	DPV	GCE	CB/DMF	0.1 M phosphate buffer pH 6.2	60	9.2 × 10^−8^ M	0.15 × 10^−6^–3.5 × 10^−6^ M	[245]
	DPV	GCE	RGO/CuTthP	0.1 M PBS pH 7.0	n/i	5.3 × 10^−9^ M	0.1–1.0 × 10^−6^ M	[250]
	LSV	GCE	Nafion	phosphate buffer pH 8.0 + CTAB	360	1 × 10^−9^ M	2.5 × 10^−8^–1.5 × 10^−6^ M	[251]
	SWV	GCE	CNT–Ni (cyclam)	0.1 M PBS pH 7.2	n/i	60 × 10^−9^ M	0.5–40 × 10^−6^ M	[252]
	amperometry	SPC	anti-estradiol-Biotin-Strept-ABAg	0.05 M PBS pH 6.0	0	2.8 × 10^–12^ M	3.7 × 10^–12^ –9.2 × 10^–10^ M	[253]
	SWV	WGE	CCh	0.1 M PBS pH 7.0	60	4 × 10^−7^ M	4 × 10^−6^–4 × 10^−5^ M	[254]
	SWV	Au	AuNPs/Protein G	0.02 M PBS pH 7.4	n/i	6.6 × 10^–12^ M	6.6 × 10^–12^ –4.4 × 10^−9^ M	[255]
	FFTSWV	CPE	(Tb_2_(CO_3_)_3_) NPs	PBS pH 5.0	n/i	3.7 × 10^–11^ M	3.7 × 10^–10^–3.7 × 10^–5^ M	[256]
	DPV	GCE	-	0.05 M H_2_SO_4_ methanol/water 9:1	n/i	1.21 × 10^−5^ M	4 × 10^−5^–1 × 10^−3^ M	[257]
	DPV	GCE	BPIDS	PBS pH 10.0	180	5.0 × 10^−8^ M	1.0 × 10^−7^–1.0 × 10^−5^ M	[258]
	DPV	CPE	GNR-FS-Au-CA	0.1 M PBS pH 5.0	180	0.0074 × 10^−6^ M	0.1–5.0 × 10^−6^ M	[259]
	LSV	GCE	RGO-DHP	0.05 M PBS pH 7.0	240	0.077 × 10^−6^ M	0.4–10 × 10^−6^ M	[260]
	SWV	HMDE	-	0.03 M B-R buffer pH 10.0	30	0.3 µg L^−1^	Up to 21.8 µg L^−1^	[261]
	DPV	CPE	Cu-BTC	phosphate buffer pH 7.0	120	0.001 × 10^−6^ M	0.003–0.75 × 10^−6^ M	[262]
	SWV	GCE	NiFe_2_O_4_-MC	0.1 M TBATFB	n/i	6.88 × 10^−9^ M	20–566 × 10^−9^ M	[263]
	SWV	GCE	Pt/MWNTs	PBS pH 7.0	180	1.8 × 10^−7^ M	5.0 × 10^−7^–1.5 × 10^−5^ M	[264]
	amperometry	CPE	FeTPyPz	0.1 M PBS pH 6.0	0	1.3 × 10^−5^ M	4.5 × 10^−5^–4.5 × 10^−4^ M	[265]
	SWV	CPE	CuO	0.1 M B-R buffer pH 9.0	n/i	21 × 10^−9^ M	60-800 × 10^−9^ M	[266]
	LSV	GCE	poly(L-serine)	0.1 M phosphate buffer pH 6.5	120	2.0 × 10^−8^ M	1.0 × 10^−7^–3.0 × 10^−5^ M	[267]
	CV	GCE	MWNT-[bmim]PF_6_	0.1 M PBS 7.0	270	5.0 × 10^−9^ M	1.0 × 10^−8^–1.0 × 10^−6^ M	[268]
	LSV	GCE	Al_2_O_3_	0.1 M phosphate buffer pH 8.0	120	8 × 10^−8^ M	4 × 10^−7^–4 × 10^−5^ M	[269]
	DPV	GCE	CdSe-BSA-antiE2	0.1 M acetate buffer pH 4.5	150	1.8 × 10^–10^ M	1.8 × 10^–10^–3.7 × 10^–10^ M	[270]
	DPV	GCE	Lac/rGO-RhNP	0.1 M PBS 7.0	n/i	0.54 × 10^−12^ M	0.9–11 × 10^−12^ M	[271]
	SWV	GCE	MWNT-Nafion	0.1 M phosphate buffer pH 7.0	300	1 × 10^–8^ M	2.5 × 10^–7^–10 × 10^–6^ M	[272]
	DPV	GC	nanosized biochar particles BCNPs	0.05 M phosphate buffer pH 4.0	1200	11.3 × 10^−9^ M	-	[273]
	DPV	SPE	CuP/ P6LC/Nafion film	0.1 M phosphate buffer pH 5.0	n/i	5 × 10^−9^ M	8 × 10^−8^–7.3 × 10^−6^ M	[274]
	SWV	BDD	CB	0.1 M phosphate buffer pH 12.0	120	2.2 × 10^−9^ M	5–100 × 10^−9^ M	[275]
	SWV	Au	PEDOT/AuNP	PBS pH 7.5	n/i	0.02 × 10^−9^ M	0.1–100 × 10^−9^ M	[276]
Estriol	SWV	CPE	Fe_3_O_4_NPs	0.1 M B-R buffer pH 6.0	-	2.7 × 10^−6^ M	3.0 × 10^−6^ –1.1 × 10^−4^ M	[277]
	DPV	GC	rGO–SbNPs	0.1 M PBS pH 9.0	30	5.0 × 10^−10^ M	2.0 × 10^−7^–1.4 × 10^−6^ M	[278]
	DPV	GC	rGO/AgNPs	0.2 M PBS pH 9.0	30	21.0 × 10^−9^ M	0.1–3.0 × 10^−6^ M	[279]
	SWV	BDD	-	0.005 M NaOH pH 12.0	n/i	1.7 × 10^−7^ M	2.0 × 10^−7^–2.0 × 10^−5^ M	[280]
	CV	CPE	PGM	0.2 M PBS pH 6.0	n/i	8.7 × 10^−7^ M	2 × 10^−6^–1 × 10^−4^ M	[281]
	DPV	GCE	CNB/AgNPs	0.1 M PBS pH 7.0	n/i	0.16 × 10^−6^ M	0.2 -3.0 × 10^−6^ M	[282]
	SWV	GCE	Pt/MWNTs	PBS pH 7.0	180	620 × 10^−9^ M	1.0 × 10^−6^–7.5 × 10^−5^ M	[264]
	CV	GCE	Ni	0.1 M NaOH pH 12.0	n/i	1.0 × 10^−7^ M	5.0 × 10^−6^–1.0 × 10^−4^ M	[283]
	LSV	GCE	RGO/GNPs/PS	0.2 M PBS pH 5.7	n/i	0.48 × 10^−6^ M	1.5–22 × 10^−6^ M	[284]
	DPV	CPE	SDS- PXAMCNTG	0.1 M PBS pH 7.0	80	1.9 × 10^−7^ M	10–70 × 10^−6^ M	[285]
	DPV	CPE	L-proline	0.1 M PBS pH 6.5	n/i	2.2 × 10^−7^ M	6 × 10^−6^-6 × 10^−5^ M	[286]
	DPV	GCE	Co-poly(Met)	0.1 M PBS pH 7.0	n/i	0.034 × 10^−6^ M	0.596-4.76 × 10^−6^ M	[287]
	CV	GCE	Ni/Co	0.1 M PBS pH 7.0	n/i	0.42 × 10^−9^ M	1 × 10^−9^–14 × 10^−9^ M	[288]
	amperometry	GCE	Lac/rGO/Sb_2_O_5_	0.1 M PBS pH 7.0	-	1.1 × 10^−8^ M	2.5 × 10^−8^–1.03 × 10^−6^ M	[289]
Estron	SWV	GCE	AuNPs-pNap	CBS pH 5.0	n/i	2.3 × 10^−13^ pg mL^−1^	3.0 × 10^−13^–2 × 10^−4^ pg mL^−1^	[290]
	SWV	WGE	CCh	0.1 M PBS pH 7.0	60	1 × 10^−7^ M	3 × 10^−7^–3 × 10^−5^ M	[254]
	SWV	BDD	-	0.25 M H_2_SO_4_	n/i	0.10 × 10^−6^ M	0.1–2.0 × 10^−6^ M	[291]
	SWV	GCE	Pt/MWNTs	PBS pH 7.0	180	840 × 10^−9^ M	2.0 × 10^−6^–5.0 × 10^−5^ M	[264]
	SWV	CPE	Fe_3_O_4_ NP-BMI.PF_6_	0.2 M B–R buffer pH 12.0	5	0.47 × 10^−6^ M	4–100 × 10^−6^ M	[292]
	SWV	CPE	-	0.1 M PBS pH 8.0	180	4.0 × 10^−8^ M	9.0 × 10^−8^–8.0 × 10^−6^ M	[293]
	LSV	GCE	MWNT/CR	0.1 M PBS pH 8.0	400	5.0 × 10^−9^ M	5.0 × 10^−8^–2.0 × 10^−5^ M	[294]
	SWV	GCE	MWCNT-COOH	B-R buffer pH 7.0	n/i	0.117 × 10^−6^ M	1.0–9.0 × 10^−6^ M	[295]
	DPV	GCE	MoSI NWs	PBS pH 7.2	n/i	5.2 × 10^−12^ g mL^−1^	2 × 10^−12^–2 × 10^−11^ g mL^−1^	[296]
Progesterone	SWV	BiFE	-	0.1 M B–R buffer pH 12.0	60	0.18 × 10^−6^ M	0.40–7.90 × 10^−6^ M	[297]
	amperometry	GCE	Mn(III)-SB	0.1 M NaOH pH 13.0	n/i	11.4 × 10^−9^ M	0.022–0.25 × 10^−6^ M	[298]
	SWV	GCE	-	0.1 M N(C_4_H_9_)_4_PF_6_ + acetonitrile	n/i	500 × 10^−9^ M	4.0–1000 × 10^−6^ M	[299]
	SWV	GCE	GO-IMZ	0.1 M NaOH pH 13.0	n/i	68 × 10^−9^ M	0.22–14.0 × 10^−6^ M	[300]
	SWV	Au	mAbP4-AuNPs	0.001 M CBS pH 5.0	n/i	0.25 × 10^−9^ M	0.0016–0.038 × 10^−6^ M	[301]
	DPV	GCE	Fe_3_O_4_@GQD/f–MWCNTs	0.1 M PBS pH 7.0	140	2.2 × 10^−9^ M	0.01–3.0 × 10^−6^ M	[302]
	DPV	GCE	Sn nanorods	0.2 M NaOH pH 13.0	-	0.12 × 10^−6^ M	40–600 × 10^−6^ M	[303]
	amperometry	SPE	mAbP4	0.1 M Diethanolamine–HCl buffer pH 9.85	-	-	0.0–0.079 × 10^−6^ M	[304]
	DPV	SPE	(prog)–BSA conjugate	milk	n/i	9.5 × 10^−9^ M	0.05–0.81 × 10^−6^ M	[305]
	DPV	CFP carbon fiber paper	CNS Carbon nanospheres	PBS pH 7.0	n/i	0.012 × 10^−9^ M	37.4 × 10^−12^–0.25 × 10^−9^ M	[306]
	SWV	nsBiFE	-	0.1 M Na-PBS	60	-	0.1– 0.7 × 10^−6^ M	[307]
	amperometry	gold-graphite-Teflon	mAbP4	0.1 M diethanolamine-HCl buffer pH 10.0	-	2.7 × 10^−9^ M	0.0–0.095 × 10^−6^ M	[308]
	amperometry	GCE	mAbP4/HRP/pyrocatechol	0.01 M PBS pH 7.0	-	0.63 × 10^−9^ M	0.0016–0.04 × 10^−6^ M	[309]
	amperometry	gold–graphite–Teflon	mAbP4	0.1 M TRIS pH 7.0 + 20 µM phenyl phosphate	-	1.4 × 10^−9^ M	0.0–0.13 × 10^−6^ M	[310]
	SWV	CPE	Gd_2_(WO_4_)_3_NPs	0.1 M B–R buffer pH 11.5	n/i	50 × 10^−9^ M	0.1–1 × 10^−6^ M	[311]
	SWV	SPE	AuNPs/AMBI/rGO	0.1 M sodium hydroxide	n/i	0.28 × 10^−9^ M	0.9 × 10^−9^–27 × 10^−6^ M	[312]
	DPV	GCE	PEDOT/ZrO_2_-NPs	CBS 0.1 M, pH 4	n/i	0.32 × 10^−9^ M	1 × 10^−9^–6 × 10^−3^ M	[313]
	DPV	SPE	BSA/aptamer/GQDs–NiO-AuNFs/f-MWCNTs	0.1 M KCl + 5.0 mM K_3_[Fe(CN)_6_]	n/i	1.86 × 10^−12^ M	0.01–1000 × 10^−9^ M	[314]
	DPV	GCE	GQD-PSSA/GO	0.1 M CBS pH 6.0	180	0.31 × 10^−9^ M	0.1–6.0 × 10^−6^ M	[315]
	DPV	GCE	PEDOT/ZrO_2_-NPs	n/i	n/i	0.32 × 10^−9^ M	1–100 × 10^−9^ M	[313]

### 3.6. Testicular Hormones

The testes are gonads found in most males of animals (except sponges). Male mammals have two testicles that are most often found in the scrotum—the fascia-dermal sac originating from the abdominal wall. In most mammals, the testes are located outside the body, suspended by a spermatic cord in the scrotum. This is because spermatogenesis is more efficient at temperatures lower than about 37 degrees Celsius inside the body. Similar to the ovaries (whose counterparts are), the testes are a component of two systems: the reproductive system (as gonads) and the endocrine system (as endocrine glands). The functions of the testicles are sperm production and the production of male sex hormones (including testosterone). Both sperm-forming and endocrine functions are under the control of the hormones produced by the anterior pituitary gland, which are lutropin (LH) and follicle-stimulating hormone (FSH) [316,317,318].

Testosterone is an organic chemical compound from the group of androgens, the basic male sex steroid hormone. It is produced by Leydig interstitial cells in the testes under the influence of the luteinizing hormone and also in small amounts by the adrenal cortex, ovaries and placenta. In the blood, only a small part of testosterone is in the free form and bound to albumin, with the rest being bound (inactive) with the SHBG transport protein (sex hormone binding globulin). In target tissues, testosterone is converted into a 2.5 times stronger form of 5-α-dihydrotestosterone. In order to exert its biological effects, testosterone binds with the receptors for steroid hormones located in the cytoplasm and nucleus of the effector cells. The treatment uses testosterone derivatives—esters for oral use or injection with a slow release from the muscle tissue. Testosterone is responsible for shaping sex and sexual characteristics in utero, spermatogenesis, development of secondary sexual characteristics, stimulating protein synthesis, increasing blood cholesterol and stimulating the development of the prostate gland, which stimulates the development of prostate cancer. Testosterone, when used in women, has an anabolic effect; however, it is very rarely practiced medicinally due to undesirable effects, such as masculinization and hirsutism. It is most often used in the case of advanced, hormonally active tumors [319,320,321].

A variety of electrochemical techniques among the different types of working electrodes were implemented for highly sensitive testosterone determination. Aside from the classic construction of the hanging mercury drop electrode, on which the testosterone limit of detection was equal, mostly solid electrodes modified with different layers and carbon paste electrodes were used. The lowest testosterone detection limit reported in the papers since 2000 was obtained using the gold electrode modified by a layer of molecularly imprinted polysiloxane thin film formed in the presence of testosterone, and it was equal to 10 fM. In the construction of other modifier layers, testosterone antibodies, carbon nanotubes, metal nanoparticles and oxides were commonly used (Table 6). In order to check the utility of the proposed methods for routine quality control analysis, the measurements of testosterone were performed in samples such as human urine, blood, plasma, serum and saliva.

In Table 6, the electrochemical methods of testosterone determination are compared.

**Table 6:** Electrochemical methods of testicular hormones determination.

Analyte	Technique	Working Electrode	Modifier	Medium	Preconcentration Time, s	Detection Limit	Linear Range	Source
Testosterone	OSWV	EPPGE	SWCNT	PBS pH 7.2	n/i	2.8 × 10^−9^ M	5–1000 × 10^−9^ M	[322]
	SWV	GCE	-	0.1 M B-R buffer pH 5.0 + 3 mM CTAB	120	1.2 × 10^−9^ M	10–70 × 10^−9^ M	[323]
	CV	GCE	CoOx	0.10 M NaOH pH 12.5	n/i	0.16 × 10^−6^ M	0.33–2.00 × 10^−6^ M	[324]
	DPV	GCE	rGO	borate buffer pH 5.4 + CTAB	n/i	0.1 × 10^−9^ M	2.0–210.0 × 10^−9^ M	[325]
	SWV	BiFE	-	0.1 M B-R buffer pH 5.0 + 3 mM CTAB	120	0.3 × 10^−9^ M	1–45 × 10^−9^ M	[326]
	DPV	CPE	MWCNT/MD	n/i	n/i	1.33 × 10^−11^ M	10 × 10^−10^–10 × 10^−8^ M	[327]
	amperometry	Teflon	antitestosterone/MWCNT/AuNPs	0.5 mM catechol+ PBS of pH 7.4 + H_2_O_2_	-	2.9 × 10^−10^ M	3.5 × 10^−9^–3.5 × 10^−8^ M	[328]
	SWV	GCE	Pb	0.05 M acetate buffer pH 5.2 + Pb(NO_3_)_2_	120	9 × 10^−9^ M	2 × 10^−8^–3 × 10^−7^ M	[329]
	SWV	Au	DMIP	0.01 M PBS pH 7.2	-	10 × 10^−15^ M	10–100 × 10^−15^ M	[330]
	amperometry	Si	α-testosterone mAb	PBS pH 7.0 + 1 mM H_2_O_2_ + 1 mM HQ	n/i	43 × 10^−12^ M	34 × 10^−12^ – 34 × 10^−9^ M	[331]
	DPV	HMDE	-	B-R buffer pH 6.5	300	5 × 10^−9^ M	1 × 10^−8^ – 7.3 × 10^−6^ M	[332]
	amperometry	SPCE	pBDBT/Ab_TES_	5.0 mM [Fe(CN)_6_]^3−/4−^ in 0.1 M KCl	n/i	17 ng mL^−1^	10–500 ng mL^−1^	[333]

### 3.7. Other Hormones and Steroids

**Figure 4:**
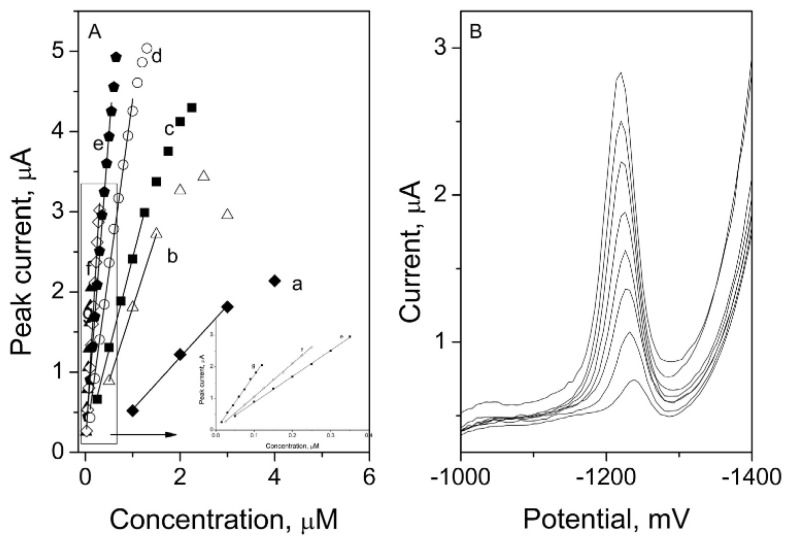
Spironolactone calibration graphs (**A**) with corresponding voltammograms for the preconcentration time of 20 s (**B**) measured on refreshable silver-based amalgam film electrode [339]. The following curves in picture A stand for preconcentration times: a: 0 s, b: 3 s, c: 5 s, d: 10 s, e: 20 s, f: 30 s and g: 45 s, measurements carried out in the 0.031 mol L^−1^ acetate buffer, pH 4.6. Reprinted from *Spironolactone voltammetric determination on renewable amalgam film electrode*, Steroids, Vol 130, Joanna Smajdor, Robert Piech, Beata Paczosa-Bator, Pages 1–6, 2018, with permission from Elsevier.

**Figure 5:**
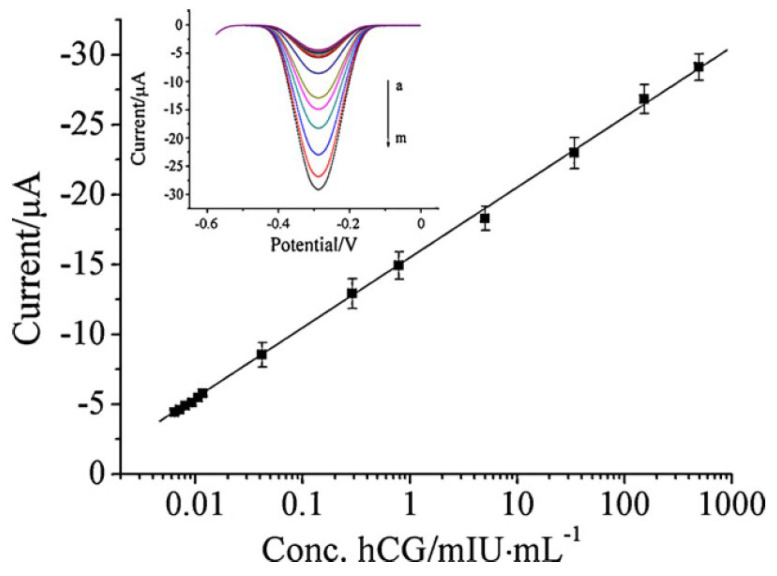
Calibration curves of the electrochemical immunosensor toward hCG standards in pH 7.0 PBS containing 2 mmol L^−1^ H_2_O_2_. Reprinted from *Ultrasensitive electrochemical immunosensor based on Au nanoparticles dotted carbon nanotube–graphene composite and functionalized mesoporous materials*, Biosensors and Bioelectronics, Vol 33, Juanjuan Lu, Shiquan Liu, Shenguang Ge, Mei Yan, Jinghua Yu, Xiutao Hu, Pages 29–35, 2012, with permission from Elsevier [340].

Aside from the previously described, the human body produces many other hormones, not only by the glands (e.g., human chorionic gonadotropin, thyroxine) but also by the adipose cells (leptin) or intestinal tract (serotonin). Thanks to modern medicine, we are able to compensate for the deficiency of the natural hormone by replacing it with an artificial one (e.g., sodium levothyroxine). Additionally, the still-growing industry of contraceptive drugs causes the development of new synthetic hormones that are similar to the natural ones (e.g., ethinylestradiol, drospirenon) [334,335,336,337,338]. Samples of the sodium levothyroxine and human chorionic gonadotropin sodium calibration graphs are presented in Figure 4 [339] and Figure 5 [340].

Another important class of biologically active compounds present in the WHO list of essential medicines is the steroid group. Steroids can be naturally produced by the organism and synthesized through chemical reactions. What they have in common is a tetracyclic carbon skeleton with 21 carbon atoms. In the human body, two classes of steroids can be distinguished: glucocorticoids and mineralocorticoids. Glucocorticoids are natural hormones that are produced by the adrenal cortex. They are of great importance in terms of metabolism, the immunological system, as well as the secretion of other hormones. The main representatives of this class of steroids are prednisone, dexamethasone or betamethasone. Mineralocorticosteroids are hormones produced in the human body by the glomerular layer of the adrenal cortex. They affect the inorganic metabolism. The main representative of mineralocorticosteroids is aldosterone. Its most important activity is the retention of sodium ions (Na^+^) in the body and the intracellular influx of potassium ions (K^+^) and the secondary retention of water in the body.

**Figure 6:**
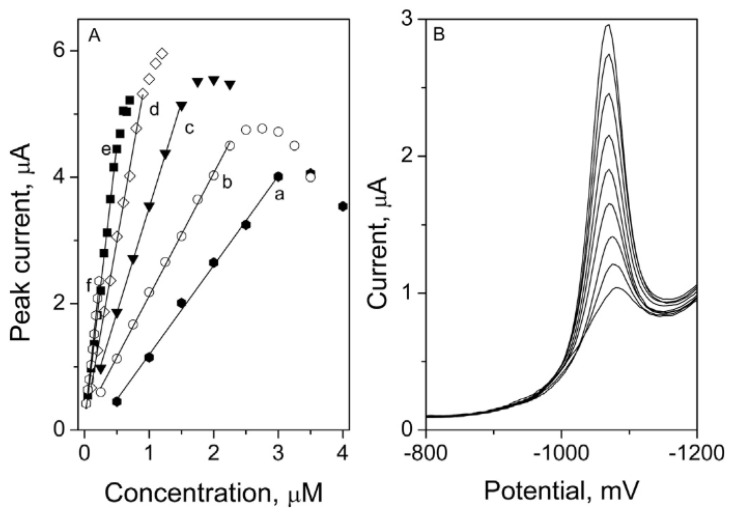
Dexamethasone calibration graphs (**A**) with corresponding voltammograms for the preconcentration time of 30 s (**B**) measured on refreshable silver-based amalgam film electrode [341]. The following curves in picture A stand for preconcentration times: a: 3 s, b: 5 s, c: 10 s, d: 20 s, e: 30 s, and f: 45 s, measurements carried out in the 0.04 mol L^−1^ acetate buffer pH 4.4. Reprinted from *Highly sensitive voltammetric determination of dexamethasone on amalgam film electrode*, Journal of Electroanalytical Chemistry, Vol 809, Joanna Smajdor, Robert Piech, Beata Paczosa-Bator, Pages 147–152, 2018, with permission from Elsevier.

**Figure 7:**
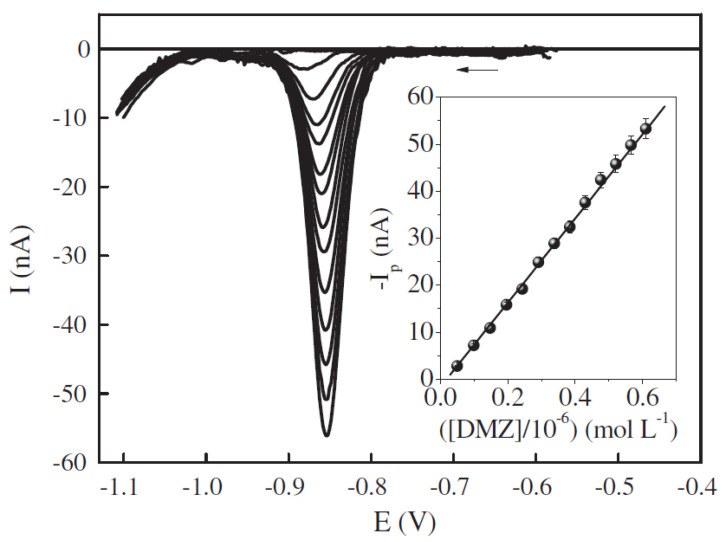
Square-wave voltammograms for DMZ in BR buffer (pH 2.0) on the HMDE with f = 100 s^−1^, a = 15 mV, dEs = 2 mV, E_acc_ = −0.60 V, t_acc_ = 15 s, and concentrations in the interval from 4.98 × 10^−8^ to 6.10 × 10^−7^ mol L^−1^ DMZ. Inset: Analytical curves obtained from voltammograms presented in the main panel. Reprinted from *Square-wave adsorptive voltammetry of dexamethasone: Redox mechanism, kinetic properties, and electroanalytical determinations in multicomponent formulations*, Analytical Biochemistry, Vol 413, Thiago Mielle B.F. Oliveira, Francisco Wirley P. Ribeiro, Janete E.S. Soares, Pedro de Lima-Neto, Adriana N. Correia, Pages 148–156, 2011, with permission from Elsevier [342].

The construction of the working electrodes in the assays of steroid determination usually comprises the solid electrode covered by the modifier layer. Additionally, classical mercury electrodes can be found in the literature, as well as carbon paste electrodes comprising carbon nanomaterials, often with the addition of nanoparticles (Table 7). Samples of the dexamethasone calibration graphs are presented in Figure 6 [341] and Figure 7 [342]. Steroids and synthetic hormones were measured in different matrices, such as human urine, serum and plasma, using voltammetric methods.

**Table 7:** Electrochemical methods of other hormones and steroid determination.

Analyte	Technique	Working Electrode	Modifier	Medium	Preconcentration Time, s	Detection Limit	Linear Range	Source
Cyproterone	SWV	GCP	AuNPs/f-MWCNT	PBS pH 5.0	30	1.7 × 10^−8^ M	9.9 × 10^−8^–1.2 × 10^−5^ M	[343]
	DDP	DME	-	B-R buffer pH 10	-	3.1 × 10^−7^ M	1.2 × 10^−6^–7.7 × 10^−5^ M	[344]
Danazol	SW-AdSV	HMDE	-	0.04 M B–R buffer pH 2	60	5.7 × 10^−9^ M	7.5 × 10^−8^–3.8 × 10^−7^ M	[345]
	DPP	DME	-	B–R buffer pH 1.0	-	1.0 × 10^−6^ M	5 × 10^−6^–1 × 10^−4^ M	[346]
Human chorionic gonadotropin	CV	GCE	GNPs/pPA/MWNTs	0.1 M PBS pH 7.0	n/i	0.3 mIU mL^−1^	1.0–160.0 mIU mL^−1^	[347]
	DPV	GCE	HRP/Ab_2_/GNPs/PB/CNTs	0.1 M PBS pH 6.0	n/i	0.023 mIU mL^−1^	0.05–150 mIU mL^−1^	[348]
	amperometry	GCE	Anti-hCG/NPG-Gs	0.067 M PBS pH 7.4	n/i	0.034 ng mL^−1^	0.5–40.00 ng mL^−1^	[349]
	amperometry	GCE	anti-hCG/Pt–Au alloy nanotube array	0.1 M PBS pH 7.5	n/i	12 mIU mL^−1^	25–400 mIU mL^−1^	[350]
	CV	GCE	anti-hCG/nano-Au/MB	0.1 M PBS pH 6.5	n/i	0.3 mIU mL^−1^	1.0–100.0 mIU mL^−1^	[351]
	Amperometry/CV	SiC	anti-HCG /Multi-layer epitaxial graphene (MEG)	PBS pH 7.4	n/i	5.62 ng mL^−1^	0.62–5.6 ng mL^−1^	[352]
	DPV	Gr–IL–Ct	PtNPs	0.1 M PBS pH 7.4 + 5 mM RU + 0.1 M KCl	n/i	0.00035 mIU mL^−1^	0.001–350 mIU mL^−1^	[353]
	amperometry	GCE	Anti-hCG/nano-gold and CS hybrid film	0.1 M PBS pH 6.5 + 0.99 mM H_2_O_2_	n/i	0.1 mIU mL^−1^	0.2–100 mIU mL^−1^	[354]
	CV	GCE	Pd–Al alloy/HRP-Ab_2_/hCG/Ab_1_/GNps/PB/GNPs	0.1 M PBS pH 7.2 + 0.8 mM H_2_O_2_	n/i	9.3 pg mL^−1^	0.5–200 pg mL^−1^	[355]
	amperometry	GCE	Anti-hCG/AuNPs	0.1 M PBS pH 6.5 + 0.99 mM H_2_O_2_	n/i	0.08 × 10^−9^ M	0.1–100 × 10^−9^ M	[356]
	DPV	GCE	Anti-hCG/Au/MWCNTs/GS	0.1 M PBS pH 7.0 + 2 mM H_2_O_2_	n/i	0.0026 × 10^−9^ M	0.005–500 × 10^−9^ M	[340]
	DPV	Au	Anti-hCG/AuNPs/Cys/AuNps	0.1 M phosphate buffer pH 7.5	n/i	0.3 pg mL^−1^	0.001–60.7 ng mL^−1^	[357]
	CV	GCE	Anti-hCG/NP-Pd/MWCNTs-BMIMPF_6_	PBS pH 7.2 + 0.2 M KCl	n/i	3.2 pg mL^−1^	0.05–50 ng mL^−1^	[358]
	amperometry	GCE	Anti-hCG/Pd@SBA-15/TH/HSO_3_-GS	PBS pH 7.4	n/i	8.6 pg mL^−1^	0.01–16.00 ng mL^−1^	[359]
	amperometry	GCE	Pt@MSNi/HRP/Ab_2_/hCG Antigene/Ab_1_/TH/Graphene	0.1 M PBS pH 7.4	n/i	7.5 pg mL^−1^	0.01–12 ng mL^−1^	[360]
	DPV	GE Graphite electrode	HRP-anti-hCG	0.1 M PBS pH 7.0	n/i	0.3 × 10^−9^ M	0.5–50 × 10^−9^ M	[361]
	DPV	GCE	BSA/anti-hCG/Au@SiC–CS	PBS pH 7.4 + 0.1 M KCl	n/i	0.042 IU L^−1^	0.1–100 IU L^−1^	[362]
	DPV	SPE	ALP-IgG labeled GNPs/Ab_2_	PBS pH 7.4	n/i	0.36 × 10^−9^ M	1.0 × 10^−9^–100.0 × 10^−6^ M	[363]
	DPV	GC	Co-poly(Met)	0.1 M PBS pH 7.0	n/i	0.113 × 10^−6^ M	0.596–4.76 × 10^−6^ M	[364]
	SWV	CPE	β-hCG-mAb/protein A	0.05 M PBS pH 7.4	n/i	15 × 10^−9^ M	30–200 × 10^−9^ M	[365]
	DPV	GCE	GS/IL/HNP-AuAg	PBS pH 7.2 + 0.2 M KCl	n/i	0.01 ng mL^−1^	0.05–35 ng mL^−1^	[366]
	DPV	GCE	HRP-anti-hCG/hCG/AuNPs-SG	0.1 M PBS pH 7.0	n/i	1.4 × 10^−9^ M	5.0–30.0 × 10^−9^ M	[367]
	DPV	CE	Au/Mab/hCGs	0.05 M PBS pH 7.4	n/i	612 × 10^−15^ M	0–2 ng mL^−1^	[368]
	amperometry	GCE	AuNPs/TB/Hb/MWNT–CS	PBS pH 6.5 + 2.1 mM H_2_O_2_	n/i	0.3 × 10^−9^ M	0.8 - 500 × 10^−9^ M	[369]
	DPV	GCE	AgNPNPs/Gr-IL-Ct	0.1 M phosphate buffer pH 7.0 + 5 mM riboflavin	n/i	0.0066 × 10^−9^ M	0.0212–530 × 10^−9^ M	[370]
	SWV	GCE	BSA/anti-hCG/CNOs/AuNPs/PEG	[Fe(CN)6]^3−/4−^	n/i	0.1 fg mL^−1^	0.1 fg mL^−1^– 1 ng mL^−1^	[371]
	DPV	GCE	Anti-hCG/AuNPs/Gr-IL-Chit	n/i	n/i	0.0016 mIU mL^−1^	0.005-411.28 mIU mL^−1^	[372]
	amperometry	Au	Hb/anti-HCG/AuNPs/{NPB/l-cys}_2_	0.025 M PBS pH 6.5	n/i	0.2 × 10^−9^ M	0.5–200 × 10^−9^ M	[373]
Leptin	DPV	GCE	SWCNTs-CT	DEA buffer solution + 0.75 mg mL^−1^ α-NP	n/i	5 pg mL^−1^	0–1000 ng mL^−1^	[374]
	SWV	GCE	PG-BP	0.1 M PBS pH 7.4	n/i	0.036 pg mL^−1^	0.150–2500 pg mL^−1^	[375]
	DPV	GCE	SWNTs/CT	DEA buffer solution + 1 mg mL^−1^ α-NP pH 9.5	n/i	30 pg mL^−1^	0.05–500 ng mL^−1^	[376]
	CV	GCE	Protein G/PPy/PPa/Au	0.01 M PBS + 1% serum pH 7.4	n/i	10 ng mL^−1^	10–100000 ng mL^−1^	[377]
	DPV	SPE	BSA/anti-LEP/EDC/NHS/MPA/Au/ Ce_3_NbO_7_/CeO_2_	0.1 M PBS pH 7.4	n/i	0.138 pg mL^−1^	0.5–12000 pg mL^−1^	[378]
	CV	ITO-PET sheets	cyanogen bromide (CNBr) anti-leptin antibody	n/i	n/i	0.0086 pg mL^−1^	0.05–100 pg mL^−1^	[379]
	DPV	SPCEs	Magnetic beads SPCE	Tris buffer+ 5% BSA pH 7.2	n/i	0.5 pg mL^−1^	5–100 pg mL^−1^	[380]
Levonorgestrel	SWV	HMDE	-	B–R buffer pH 3	150	4.8 × 10^−10^ M	1 × 10^−9^–1 × 10^−7^ M	[381]
	SWV	solid amalgam electrode	AgNPs	0.04 M B-R buffer pH 6.0.	70	9.09 × 10^−8^ M	5.0 × 10^−7^–1.0 × 10^−5^ M	[382]
Methylprednisolone	DPV	GCE	fullerene-C_60_	0.5 M PBS pH 7.2	n/i	5.6 × 10^−9^ M	5.0 × 10^−9^–1.0 × 10^−6^ M	[383]
	SWV	EPPGE	SWNTs	1.0 M PBS pH 7.2	n/i	4.5 × 10^−9^ M	5–500 × 10^−9^ M	[384]
	DPV	ITO	AuNPs	0.5 M PBS pH 7.2	n/i	2.7 × 10^−7^ M	0.01–1.0 × 10^−6^ M	[385]
Nandrolone	SWV	GCE	fullerene-C_60_	0.05 M PBS pH 7.2	n/i	0.42 × 10^−9^ M	50 × 10^−6^–0.1 × 10^−9^ M	[386]
	OSWV	EPPGE	-	0.05 M PBS pH 7.2	n/i	1.5 × 10^−11^ M	0.01–50 × 10^−9^ M	[387]
	DPV	ITO	AuNPs	PBS pH 7.2	n/i	1.4 × 10^−7^ M	50 × 10^−9^–1.5 × 10^−6^ M	[388]
Oxytocin	amperometry	BDD	-	Tris buffer pH 7.4	n/i	50 × 10^−9^ M	0.1–10 ^×^ 10^−6^ M	[389]
Serotonin	DPV	GCE	MWCNT	phosphate buffer pH 7.0	120	5 × 10^−9^ M	2 × 10^−8^–5 × 10^−6^ M	[390]
	DPV	GCE	choline	0.1 M PBS pH 4.0	n/i	5 × 10^−7^ M	1 × 10^−6^–3 × 10^−5^ M	[391]
	OSW	CPE	iron(II) phthalocyanine complexes [FeTSPc]^4−^	pH 7.4	n/i	1 × 10^−6^ M	1 × 10^−6^ –1.51 × 10^−5^ M	[392]
	SWV	GCE	C-undecylcalix [4]resorcinarene (CUCR)	0.2 M phosphate buffer pH 7.0	120	3.0 × 10^−8^ M	1.0 × 10^−7^–1.0 × 10^−5^ M	[393]
	DPV	graphite	MWCNT	50 mM phosphate buffer pH 7.4	n/i	0.2 × 10^−6^ M	1.0–15 ^×^ 10^−6^ M	[394]
	SWV	ITO	AuNPs	0.1 M PBS pH 7.2	n/i	3.0 × 10^−9^ M	1.0 × 10^−8^–2.5 × 10^−4^ M	[395]
	SWV	EPPG	polymelamine	PBS pH 7.2	n/i	30 × 10^−9^ M	0.1–100 × 10^−6^ M	[396]
	DPV	GCE	nano-Au/PPyox	0.1 M PBS pH 7.0	240	1.0 × 10^−9^ M	7.0 × 10^−9^–2.2 × 10^−6^ M	[397]
	DPV	GCE	reduced graphene oxide (RGO)	0.1 M PBS pH 7.4	n/i	3.2 × 10^−8^ M	1.0 × 10^−6^–1.0 × 10^−4^ M	[398]
	CV	pencil graphite electrode (PGE)	poly(pyrrole-3-carboxylicacid) (p(P3CA)	0.1 M PBS pH 5.0	90	2.5 × 10^−9^ M	0.01–1.0 × 10^−6^ M	[399]
	DPV	Pt	MWCNT/PPy/AgNPs	0.2 M PBS pH 8.0	n/i	0.15 × 10^−6^ M	0.50–5.0 × 10^−6^ M	[400]
	DPV	GCE	poly(phenosafranine)	0.1 M PBS pH 7.1	n/i	20 × 10^−9^ M	-	[401]
	amperometry	BDD	-	0.1 M phosphate buffer pH 7.0	-	10 × 10^−9^ M	10 × 10^−9^ –50 × 10^−6^ M	[402]
	SWV	Au	acetylene black nanoparticles-chitosan (AB-C)	0.1 M PBS pH 7.0	0	1.6 × 10^−7^ M	5 × 10^−7^–1.0 × 10^−4^ M	[235]
	SWV	GCE	AuNPs	0.2 M PBS pH 7.0	n/i	2.0 × 10^−8^ M	6.0 × 10^−8^–6.0 × 10^−6^ M	[403]
	DPV	GCE	5-hydroxytryptophan (5-HTP)	0.1 M PBS pH 6.0	n/i	1.7 × 10^−6^ M	5.0 × 10^−6^–3.5 × 10^−5^ M	[404]
	DPV	GCE	MWNTs-MO-Gel	0.1 M phosphate buffer pH 7.0	240	8.0 × 10^−8^ M	20 × 10^−9^–7 × 10^−6^ M	[405]
Triamcinolone	SWV	GCE	-	0.1 M KOH pH 13.0	60	2.5 × 10^−8^ M	3.8 × 10^−8^–1.2 × 10^−4^ M	[406]
	OSWV	EPPG	SWNTs/fullerene-C_60_	phosphate buffer pH 7.2		8.9 × 10^−10^ M	0.1-25 × 10^−9^ M	[407]
Thyroxine	DPV	Ag	1, 3 diacryl urea, (DAU)	PBS pH 6.0	150	7.7 × 10^−12^ M	1.3 × 10^−11^–2.2 × 10^−8^ M	[408]
	CV	CPE	PVP	0.1 M NaOH + CTAB pH 13.0	300	8 × 10^−8^ M	2 × 10^−7^–9 × 10^−6^ M	[409]
	LSV	GCE	SWNT	0.1 M NaOH + CTAB pH 13.0	120	2 × 10^−8^ M	1 × 10^−7^–7 × 10^−6^ M	[410]
	CV	CPE	-	0.1 M HCl + CTAB	300	6.5 × 10^−9^ M	2 × 10^−7^–9 × 10^−6^ M	[411]
	DPV	GCE	MWCNT	0.1 M HCl	120	6.4 × 10^−9^ M	1.9 × 10^−8^–5.1 × 10^−7^ M	[412]
	CV	CPE	AuNPs/rGO	0.1 M HCl	n/i	1.0 × 10^−9^ M	1.0–14.0 × 10^−9^ M	[413]
Mometasone	SWV	GE	SWCNT	0.1 M phosphate buffer pH 7.2 + CTAB	-	1.23 × 10^−6^ M	10–1000 × 10^−6^ M	[414]
Dienogest	DPP	DME	-	phosphate buffer pH 10.8	-	0.58 × 10^−6^ M	2.0 × 10^−6^ –1.0 × 10^−4^ M	[415]
	SWV	GCE	ERGO/f-MWCNT/AuNPs	0.1 M PB solution pH 3.0	60	1.88 × 10^−8^ M	2.0 × 10^−7^–6.0 × 10^−6^ M	[416]
Drospirenone	DPV	Hg(Ag)FE	-	Acetate buffer pH 6.0 + Marlinat	60	1.7 × 10^−9^ M	2.5 × 10^−9^–1.0 × 10^−8^ M	[417]
	SWV	HMDE	-	0.04 M B-R buffer pH 8.0	30	0.027 µg mL^−1^	1.36 × 10^−6^–1.91 × 10^−7^ M	[418]
Finasteride	DPP	CGME	-	Ethanol + 0.0625 M H_2_SO_4_ pH<2	n/i	7.59 × 10^−6^ M	5 × 10^−5^–5 × 10^−4^ M	[419]
Spironolactone	DPV	Hg(Ag)FE	-	0.03 M acetate buffer pH 4.6	45	4.7·10^−9^ M	15·10^−9^–3.0·10^−6^ M	[339]
	DPV	HMDE	-	B-R buffer pH 9.0	60	1.1 × 10^−11^ M	1.2 × 10^−10^–9.6 × 10^−7^ M	[420]
	SWV	CPE	AuNPs@FCBN-PE	PBS pH 4.0	60	3.34 × 10^−9^ M	12–1200 × 10^−9^ M	[421]
	DPV	HMDE	-	B-R buffer pH 2.5	90	1.72 × 10^−10^ M	1 × 10^−8^–2.5 × 10^−7^ M	[422]
Ethinylestradiol	DPV	GCE	CB/DMF	0.01 M borate buffer pH 10.0	20	0.13 × 10^−6^ M	0.25 × 10^−6^–3.0 × 10^−6^ M	[423]
	SWV	GCE	MWCNTs	10 mM H_2_SO_4_ pH 2	60	3.4 × 10^–14^ M	1.2 × 10^–13^–2.4 × 10^–10^ M	[424]
	amperometry	GCE	AgNPs/SiO_2_/GO/anti-EE2	0.1 M PBS pH 7.2 + 45 µL1 mM HQ + 5 µL 50 mM H_2_O_2_	0	2.2 × 10^–11^ M	3.4 × 10^–11^-1.7 × 10^–7^ M	[425]
	SWV	HMDE	-	B–R buffer pH 7.0	60	5.9 × 10^−10^ M	2.9 × 10^−9^–5 × 10^−7^ M	[426]
	LSV	CPE	-	0.07 M phosphate buffer pH 8.04 + CPB	150	3.0 × 10^−8^ M	5.0 × 10^−8^–2.0 × 10^−5^ M	[427]
	SWV	FTO	Chi/CNTs	0.01 M PBS pH 7.0	150	0.09 × 10^−6^ M	0.05–20 × 10^−6^ M	[428]
	DPV	HMDE	-	0.03 M B-R buffer pH 10.0	30	9.7 µg L^−1^	Up to 400.5 µg L^−1^	[261]
	DPV	HMDE	-	0.04 M B-R buffer pH 8.0	30	3.58 ng mL^−1^	6.75 × 10^−8^–6.07 × 10^−7^ M	[418]
	SWV	GCE	MWCNTs-CoPc	0.1 M phosphate buffer pH 7.0	n/i	2.2 × 10^−6^ M	2.5–90 × 10^−6^ M	[429]
	DPV	HMDE	-	B–R buffer pH 7.0	150	1.7 × 10^–9^ M	1.3 × 10^–8^–2.0 × 10^–7^ M	[430]
	SWV	BDD	-	0.1 M B-R buffer pH 8.0	n/i	2.4 × 10^−7^ M	9.9 × 10^−7^–5.2 × 10^−6^ M	[431]
	DPV	GCE	rGO/RuO_2_	0.1 M PBS pH 7.0	n/i	2.0 × 10^−9^ M	5.5 × 10^−8^ –1.2 × 10^−6^ M	[432]
Prednisolone	DPV	Hg(Ag)FE	-	0.075 M acetate buffer pH 3.8	20	0.01 × 10^−6^ M	0.05 × 10^−6^–2.25 × 10^−6^ M	[433]
	amperometry	FTO	α-NiCe	1.0 M KOH	0	8.4 × 10^−9^ M	0.5–76.9 × 10^−6^ M	[434]
	OSWV	EPPG	SWNT	Phosphate buffer pH 7.2	n/i	0.45 × 10^−8^ M	0.01–100 × 10^−6^ M	[435]
	DPV	Au	Fullerene C_60_	0.1 M PBS pH 7.2	n/i	26 × 10^−9^ M	1 × 10^−6^–0.1 × 10^−3^ M	[436]
	DPV	HMDE	-	0.04 M B-R buffer pH 3.5	40	1.1 × 10^–7^ M	2.0 × 10^–7^–4.0 × 10^–7^ M	[437]
	DPV	CPE	β-cyclode × trin	B-R buffer pH 3.0	150	4.8 × 10^−7^ M	5.6 × 10^−7^–2 × 10^−5^ M	[438]
	OSWV	EPPG	Fullerene C_60_	PBS pH 7.2	n/i	4.8 × 10^−8^ M	0.05–50 × 10^−6^ M	[439]
	SWV	GC	OMC	0.05 M PBS pH 7.2	n/i	0.057 × 10^−6^ M	0.06–40 × 10^−6^ M	[440]
	DPV	MIP	MWCNT	Phosphate buffer pH 4.0	60	0.05 × 10^−6^ M	0.08–160 × 10^−6^ M	[441]
	DPV	HMDE	-	B-R buffer pH 3.78	n/i	1.6 × 10^–8^ M	5.6 × 10^–8^ –1.1 × 10^–6^ M	[442]
	SWV	GCE	carbon nano sphere (CNSs) modified	0.1 M PBS pH 7.2	n/i	73 × 10^−9^ M	3-50 × 10^−6^ M	[163]
Betamethasone	DPV	Hg(Ag)FE	-	0.1 M acetate buffer pH 3.0	45	1.6 × 10^−9^ M	5.0 × 10^−9^ –0.8 × 10^−6^ M	[443]
	SWV	EPPG	SWNT	1 M PBS pH 7.2	n/i	0.25 × 10^−9^ M	0.5–100 × 10^−9^ M	[444]
	OSWV	EPPG	SWNT	1 M PBS pH 7.2	n/i	0.5 × 10^−9^ M	1–25 × 10^−9^ M	[445]
	DPP	HMDE	-	0.04 M B-R buffer pH 1.7	n/i	2.7 × 10^−6^ M	3.9 × 10^–6^–1.1 × 10^–4^ M	[446]
Sodium levothyroxine	DPV	Hg(Ag)FE	-	0.1 M sodium tetraborate solution + 300 µl HCl (1:10) pH 2.3	30	1.8 × 10^−8^ M	0.025 × 10^−6^–4.0 × 10^−6^ M	[447]
	DPV	SPE	CNT	0.1 M acetate buffer pH 4.0	300	30 × 10^−9^ M	0.1–0.9 × 10^−9^ M	[448]
	CV	CPE	-	0.1 M HCl + 0.1 mM phenyl hydrazine	n/i	2.5 × 10^−6^ M	0.025–0.1 × 10^−3^ M	[449]
	CV	CPE	-	0.1 M HCl	n/i	n/i	2 × 10^−4^–2.2 × 10^−3^ M	[450]
	DPV	GCE	MWCNTs/CC-SH/Au	PBS pH 7.2	n/i	2.84 × 10^−9^ M	10–120 × 10^−9^ M	[451]
Dexamethasone	DPV	Hg(Ag)FE	-	0.04 M acetate buffer pH 4.4	45	1.6 × 10^−9^ M	2.5 × 10^−9^ –2.3 × 10^−7^ M	[341]
	SWV	HMDE	-	0.04 M B-R buffer pH 2.0	15	2.54 × 10^−9^ M	5.0 × 10^−8^ –6.1 × 10^−7^ M	[342]
	SWV	PGE	Fullerene C_60_	PBS pH 7.2	n/i	5.5 × 10^−8^ M	0.05–100 × 10^−6^ M	[452]
	SWV	HMDE	-	0.04 M B-R buffer pH 2.0	15	2.54 × 10^–9^ M	7.5 × 10^–9^–1.8 × 10^–8^ M	[453]
	DPV	CPE	Β-cyclodextrin	B-R buffer pH 3.0	150	3.6 × 10^–7^ M	4.1 × 10^–7^–2 × 10^–5^ M	[163]
	DPV	CFMS	-	0.1 M PBS pH 7.3	n/i	4 × 10^−9^ M	10 × 10^−9^–40 × 10^−6^ M	[454]
	DPV	CILE	Fe_3_O_4_/PANI–Cu	0.1 M KH_2_PO_4_ pH 2.0	n/i	3.0 × 10^–9^ M	0.05–30 × 10^−6^ M	[455]
	DPP	HMDE	-	Acetate buffer pH 5.0	0	7.6 × 10^−6^ M	25.5–122.3 × 10^−6^ M	[456]
	LSV	CPE	polyglycine-MWCNTs	B-R buffer pH 3.0	60	2.2 × 10^–7^ M	4.8 × 10^–7^–4.9 × 10^–5^ M	[457]
	SWV	Au	DNA aptamer	0.01 M PBS buffer pH 7.4	n/i	2.12 × 10^−9^ M	2.5–100 × 10^−9^ M	[458]
	DPV	Nano-porous GCE	-	0.1 M PBS pH 2	n/i	5 × 10^−9^ M	0.02–22 × 10^−6^ M	[171]
	DPV	GCE	Graphene nanoplate GNP	0.1 M PBS pH 7.3	n/i	15 × 10^–9^ M	0.1-5000 × 10^–6^ M	[459]
	SWV	EPPGE	SWNT	phosphate buffer pH 7.2	n/i	9.1 × 10^−10^ M	8.2 × 10^−8^–9.1 × 10^−10^ M	[460]
	SWV	PE	MWCNTs	Acetate buffer pH 4.0	30	0.09 × 10^−6^ M	0.15–100 × 10^−6^ M	[461]

## 4. Future Trends

There is still a growing interest in designing a new construction of the working electrodes to prepare new measurement assays and obtain lower limits of detection. There is still a wide variety of possible modifier combinations that may result in discovering new analytical assays of hormones in steroids. Specifically, the field of immunosensors is very promising for this case, considering the possibilities of obtaining low detection limits by using specific antibodies for the selected analyte. Additionally, increased interest in the construction of the new hybrid composites of modifier layers is promising, considering the new possibilities of developing voltammetric assays for highly sensitive hormone and steroid determinations. The choice of electrode modifier is always connected with the possibility of improving the sensitivity of the measurements. One way of making such an improvement is by increasing the working surface of the working electrode, which is a natural consequence of nanomaterials usage in the modifier layer. Another way of improving the sensitivity of the measurements is the impact of the electrocatalytic properties of the modifiers. Hybrid materials are nowadays gaining more interest as electrode modifiers due to the possibility of mixing the properties of different components. Modifiers, such as metal nanoparticles or metal oxides, positively affect the easiness of electron transference during the electrode process, which influences the current response of the analyte. Modifiers’ compositions of the nanoparticles mixed with carbon nanomaterials can be described as the increased density of active sites and large surface area, which ensures multiple absorption active sites and better electrical conductivity in comparison with the unmodified electrode. However, the use of electrochemical techniques for the highly sensitive hormone and steroid determination may also be limited in the case of analyses of non-electroactive compounds. Additionally, an analysis of the real samples, such as human body fluids, may be troublesome due to its complex organic matrix, which may interfere with the measurements and influence the lower sensitivity of the detection. However, considering all the above, it can be said that the voltammetric techniques can be useful in the context of pharmaceutical formulation quality control.

## 5. Conclusions

Numerous cases of voltammetric measurements of electroactive samples with biological significance, such as hormones and steroids, have been reported since 2000. The voltammetric and polarographic techniques, due to their sensitivity and easiness, could be used alternatively to other, more complicated analytical assays. The process of sample preparation for electrochemical measurements is usually quick and does not require expensive procedures. Moreover, the mechanisms of analyte reactions on the surface of a working electrode and its redox properties, which can be investigated using the cyclic voltammetry technique, could provide additional knowledge about the interaction of the compound with cells and tissues. It has also been proven that electrochemical techniques are useful for hormone and steroid determination, not only in a simple supporting electrolyte but also in more complex matrices, such as tablets, ointments, creams and different biological fluids.

## Data Availability

No new data were created or analyzed in this study. Data sharing is not applicable to this article.
